# The Evolution of Different Forms of Sociality: Behavioral Mechanisms and Eco-Evolutionary Feedback

**DOI:** 10.1371/journal.pone.0117027

**Published:** 2015-01-28

**Authors:** Daniel J. van der Post, Rineke Verbrugge, Charlotte K. Hemelrijk

**Affiliations:** 1 Institute of Artificial Intelligence, University of Groningen, Groningen, The Netherlands; 2 Behavioural Ecology and Self-Organization, University of Groningen, Groningen, The Netherlands; 3 Centre for Social Learning and Cognitive Evolution, University of St. Andrews, St. Andrews, Fife, United Kingdom; Peking University, CHINA

## Abstract

Different forms of sociality have evolved via unique evolutionary trajectories. However, it remains unknown to what extent trajectories of social evolution depend on the specific characteristics of different species. Our approach to studying such trajectories is to use evolutionary case-studies, so that we can investigate how grouping co-evolves with a multitude of individual characteristics. Here we focus on anti-predator vigilance and foraging. We use an individual-based model, where behavioral mechanisms are specified, and costs and benefits are not predefined. We show that evolutionary changes in grouping alter selection pressures on vigilance, and vice versa. This eco-evolutionary feedback generates an evolutionary progression from “leader-follower” societies to “fission-fusion” societies, where cooperative vigilance in groups is maintained via a balance between within- and between-group selection. Group-level selection is generated from an assortment that arises spontaneously when vigilant and non-vigilant foragers have different grouping tendencies. The evolutionary maintenance of small groups, and cooperative vigilance in those groups, is therefore achieved simultaneously. The evolutionary phases, and the transitions between them, depend strongly on behavioral mechanisms. Thus, integrating behavioral mechanisms and eco-evolutionary feedback is critical for understanding what kinds of intermediate stages are involved during the evolution of particular forms of sociality.

## Introduction

Understanding how different forms of sociality evolve is a major challenge. While in many cases, sociality has probably evolved due to common evolutionary pressures, such as predation [1–3], the evolutionary consequences of grouping differ across taxonomic groups. For instance, compared to antelope, primates typically have stronger social bonds and greater relative brain size [4, 5]. Differences between these two taxonomic groups probably depend on how grouping has co-evolved with other aspects of biology such as morphology, diet, defense of resources, life-history parameters, and cultural inheritance [6–10]. It is a challenge to find out to what extent specific characteristics of species affect trajectories of social evolution. We therefore use multi-scale models to investigate how grouping co-evolves with other aspects of a species’ biology.

Two factors are likely to be important for the co-evolution of grouping with other biological traits. First, such co-evolution involves multiple social phenomena, which are inter-related. Using models, it has been shown that grouping can interact (i) with diet learning, to generate cultural phenomena [11, 12]; (ii) with dominance interactions and grooming, to generate various social patterns [13–16]; and (iii) with anti-predator vigilance, to generate detection of predators at a group level [17, 18]. The grouping process is therefore causally related to multiple social phenomena, and as a result, evolutionary changes in one phenomenon can cause changes in another.

Second, the co-evolution of multiple social phenomena may generate eco-evolutionary feedback. Here, eco-evolutionary feedback refers to the process where the evolution of a trait, such as grouping, changes social and/or ecological conditions, resulting in changes in the selective pressures on other traits, such as vigilance, and vice versa. Such reciprocal feedback is referred to as (social) niche construction [19, 20], and can operate on at least two timescales.

On relatively short timescales, eco-evolutionary feedback can be expected to alter the socio-ecological dynamics of particular forms of sociality, as compared to dynamics without evolution. For instance, in the context of predator-prey co-evolution, model studies have revealed that eco-evolutionary feedback can affect the number of predator and prey species that can co-exist, as well as shortening the period of population dynamics [21]. On relatively long timescales, eco-evolutionary feedback is a prerequisite for the origin and evolution of new forms of sociality: Evolution changes the selection pressures and therewith the prevailing social phenotypes and their socio-ecological relationships that characterize a social system. The analogy with evolutionary game theory, where socio-ecological conditions are simplified into games with a set of strategies and payoffs, is that eco-evolutionary feedback alters the dynamics within games on short timescales, and changes one game into another on longer timescales (e.g. one with or without a cooperative dilemma). The long-term process concerns shifts in functionality of traits, such as most likely occurred in primates, who groom much more frequently than would be expected for its original purpose of hygiene [22]: It is possible that during the evolution of grouping in primates, evolutionary pressures on grooming were altered, and grooming became co-opted for social bonding, enabling new kinds of sociality to evolve.

**Current theory on co-evolution of social phenomena:** At present, theory on evolutionary trajectories with changing forms of sociality and shifts in functionality of traits, is highly underdeveloped. It is mostly unknown how evolutionary trajectories depend on (i) mechanistic details, (ii) the co-evolution of multiple social traits, and (iii) eco-evolutionary feedback. In many models, mechanistic details are not specified [23] and the focus is on particular social traits in isolation, such as optimal group size [24–27], optimal vigilance levels in groups [17, 28–30], or strong abstractions such as cooperative strategies with fixed payoffs [31–33]. In such models, evolution is constrained to fixed socio-ecological settings (‘games’).

However, a growing body of evidence suggests that the inclusion of mechanistic detail [34, 35], co-evolution of social traits [36, 37], and eco-evolutionary feedback [20, 21, 38, 39], leads to qualitatively different model outcomes. In particular, recent models in which grouping and cooperation co-evolve [36, 37], reveal the importance of multi-level selection and evolutionary change in the interaction structure of a population: Group sizes evolve to the point where between-group selection can maintain cooperation within populations. Since there are many social traits that could co-evolve, such results may be just the tip of an iceberg.

**Evolutionary case-studies:** One way of studying the co-evolution of multiple traits and the evolution of socio-ecological settings is to use evolutionary case-studies. Here we conduct such a case-study in a model where grouping co-evolves with foraging, movement, and anti-predator vigilance behavior. In our model, simplistic foragers search for food, and can detect predators and escape from them. Predators search for and attempt to catch foragers (S1 Video). This occurs in a 2-dimensional spatial environment, where 2 predators and roughly 100 foragers move in a continuous space. A set of evolvable parameters determines grouping tendencies, and the propensity of foragers to scan for predators. If grouping tendencies evolve, contagious fleeing transmits information about predators within groups (S2 Video). Vigilance and grouping both lead to reduced predation risk and longer lifespan, but reduce the rate of food intake and reproduction: Grouping generates within-group competition and vigilance takes time and trades off with foraging. Since foragers can benefit from the vigilance of others, this sets the stage for a cooperative dilemma and within-group selection against vigilance.

We start our simulations with solitary individuals and study what kinds of sociality evolve. We do this to address the following theoretical issues: (i) How does cooperative foraging originate via the co-evolution of vigilance and grouping tendencies? (ii) Does eco-evolutionary feedback arise after the origin of grouping, and what are its consequences? (iii) How does an evolutionary trajectory depend on mechanistic detail and the co-evolution of multiple traits?

Our results show that group foraging evolves because transmitting information about predators in groups generates synergy. Moreover, we find that mechanistic detail determines the nature of the co-evolution of multiple traits, and results in an eco-evolutionary succession of distinct forms of sociality, namely leader-follower societies characterized by a ‘traveling mutualism’, which is followed by dynamic fission-fusion societies, characterized by a ‘cooperative dilemma’. In the latter societies, a co-evolution between grouping tendencies and vigilance leads to the evolution of group sizes where cooperative vigilance can be maintained.

## Materials and Methods

The aim of our approach is to capture the multi-scale nature of behavioral processes and patterns, as well as their ecological and evolutionary consequences [40–43], so that socio-ecological settings (i.e. strategies, and their costs and benefits) are not predefined. Our model is therefore designed so that sociality is an emergent property that self-organizes from the interaction of individuals with their local environment.

In our individual-based model, we specify (i) the size of the environment and the initial distribution, energy content and renewal of resources, (ii) the number of predators and how easily they catch foragers, (iii) the local sensing and decision-making of foragers and predators (local information processing), and (iv) parameters that define how much energy foragers obtain from resource items, and the energy they require to reproduce (life-history constraints). The specifications in our model define the relationship between the environment (32 km^2^), maximum life-span (20 years), minimum inter-birth interval (several months), detection ranges (<50 meters), duration of behavioral actions (>10s), and maximal movement speed (0.1 m/s). This spatio-temporal scaling context is inspired by, and is probably most relevant to, small mammals such as monkeys, but also takes computational constraints into account.

**Figure 1:**
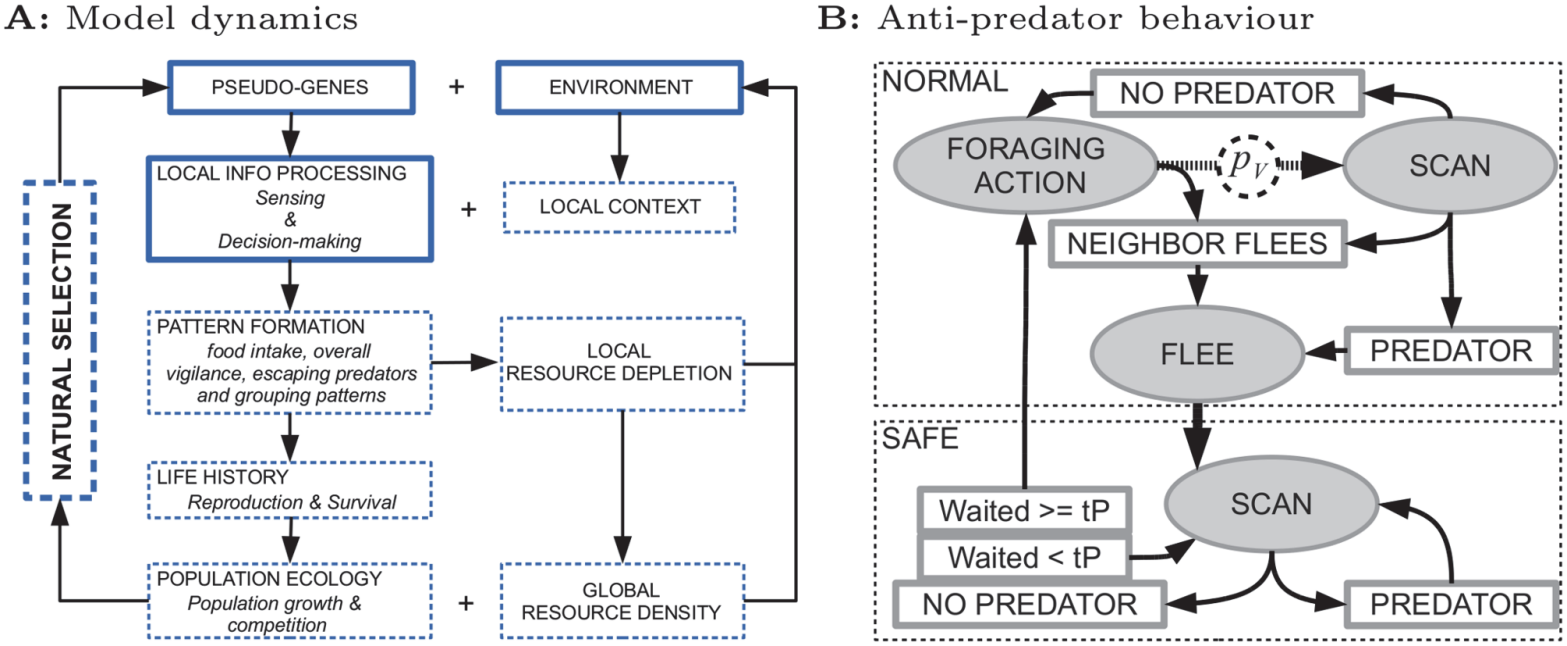
Summary of model dynamics and anti-predator behavior. A) Model dynamics. Solid boxes indicate model aspects that are implemented, namely the initial environment and pseudo-genes defining sensing and decision making of foragers. Dashed boxes indicate emergent processes on longer timescales: As foragers interact with local contexts in the environment, they generate behavioral patterns which in turn determine reproduction and survival and cause resource depletion. Reproduction and survival affect population growth and competition. If variation in pseudo-genes (due to mutations) results in differential reproduction and survival, natural selection emerges. B) Anti-predator behavior. Foragers start in a normal foraging state (top dashed box) and can decide to scan for a predator with probability *p*_*V*_. Only if a predator is detected, or a neighbor flees, does the forager flee, at which point it reaches a safe state (bottom dashed box). Here it continues to scan until it no longer detects the predator. After that, the forager continues scanning for another *t*_*P*_ minutes, before it returns to a normal foraging state.

From these specifications (Fig. 1A, solid boxes), various dynamics emerge on multiple timescales (Fig. 1A, dashed boxes). The most immediate dynamic is behavioral pattern formation: the interaction between decision-making and local environmental context generates behavioral patterns, such as foraging, vigilance and grouping. Food intake rates then determine at what rate foragers reproduce or whether they starve, and vigilance and grouping patterns determine survival via predation avoidance. In turn, these life-history variables determine population growth and competition. Competition arises from depletion of resources in the environment, and the population grows to the point where births and deaths are balanced on average (90–120 foragers, depending on vigilance levels). The population density (3.75 individuals per km^2^) is then within the range observed for monkeys [44]. During reproduction, mutations can change the value of the pseudo-genes (parameters) that define sensing and decision making of foragers (Table 1). If genetic differences give rise to differences in reproduction and survival, this leads to natural selection for pseudo-genes that generate the most successful behavioral patterns.

**Table 1:** Evolvable parameters of foragers.

**Category**	**Parameter**	**Min**	**Max**	**St. dev.**	**Units**
Decision making (probability to choose action)	*p*_*V*_ (predator scan)	0.0	1.0	0.2	
	*p*_*M*_ (move after move)	0.0	1.0	0.2	
	*p*_*SE*_ (food scan after eat)	0.0	1.0	0.2	
	*p*_*SN*_ (food scan after nofood)	0.0	1.0	0.2	
	*p*_*MTF*_ (move to food)	0.0	1.0	0.2	
Vigilance	*t*_*V*_ (scan duration)	0.167	1.99	0.4	min
	*a*_*V*_ (scan angle)	0.0	360	72	degrees
Fleeing	*t*_*P*_ (flee duration)	0.0	-	5	min
Grouping	*a*_*R*_ (repulsion turning angle)	0.0	360	72	degrees
	*a*_*A*_ (attraction turning angle)	0.0	360	72	degrees
	*z*_*R*_ (repulsion zone)	0	50	10	m
	*z*_*A*_ (attraction zone)	*z*_*R*_	50	10	m
	*n*_*R*_ (tolerated neighbors)	0	-	1	m
Movement	*t*_*M*_ (move duration)	0.167	1.99	0.4	min
	*d*_*M*_ (move distance)	0.0	-	3	m
	*a*_*M*_ (move angle)	0.0	360	72	degrees
Foraging	*t*_*F*_ (foodscan duration)	0.167	1.99	0.4	min
	*d*_*F*_ (foodscan distance)	0.0	-	3	m
	*a*_*F*_ (foodscan angle)	0.0	360	72	degrees

The standard deviation of mutation is scaled to be approximately 0.2 times the range of the parameter. The maximum of durations was imposed due to how the model was programmed, but was high enough not to affect the results.

Darwinian fitness therefore depends on how behavior patterns affect survival and reproduction of individuals, and overlapping generations ensure that Darwinian fitness is relative to an existing population structure. Since all social phenomena (e.g. coordinated travel and vigilance in groups) emerge through grouping, they are causally related. This enables eco-evolutionary feedback, where newly evolved social phenomena, such as grouping, can alter selection pressures on other behavior, such as vigilance, so that socio-ecological settings (‘games’) can evolve.

The high spatio-temporal resolution requires simplification of social, mechanistic and environmental detail. Simplifications include low cognitive sophistication of individuals, low environmental diversity, and a limit on the number of social traits. Such simplifications can be addressed by using relatively basic case-studies as baselines, so that on their basis, the dynamics in more complex case-studies can be understood. The present study therefore increases the complexity of our evolutionary system relative to previous studies on the evolution of solitary and group foraging without predation [43, 45]. Below we describe individual behavior, life-history, and the environment in more detail.

**Local information processing:** The behavioral repertoire of foragers is implemented as a set of actions that take time: (i) foraging: MOVE, FOODSCAN, MOVETOFOOD, EAT; (ii) anti-predation: SCAN and FLEE (Fig. 1B). We use an event-based formalism where action durations can evolve to minimally 10 seconds. Foraging is initialized using previously evolved parameters, which have been described and extensively studied elsewhere [43]. Although foraging parameters can evolve in the present study, they do not qualitatively change (see Supporting Information S2 Text). Since foraging and vigilance actions take time, there is a trade-off between vigilance and foraging.

*Vigilance:* Evolvable vigilance parameter *p*_*V*_ determines the rate at which foragers SCAN for predators after each foraging action (Fig. 1B). The probability to detect the predator *p*_*D*_ depends on distance (as in [18]): $p_{D}=\alpha (h^{N}/[d_{FP}^{N}+h^{N}])$ where *N* determines how steeply predator detection decreases with distance to the predator *d*_*FP*_ (maximally 50 meters), *h* is the distance at which predator detection probability is half maximal, and *α* = 0.5. If they detect a predator, foragers FLEE and escape. A forager also flees if a neighbor flees, or if a predator attacks. After FLEE, foragers reach safety, and are subsequently ignored by the predator (Fig. 1B, bottom dashed box). During FLEE, foragers do not change position, like monkeys that rush up a tree at a given location (S2 Video). After FLEE, foragers continue to detect the predator as long as the predator is within 50 meters. Thereafter, foragers continue to SCAN for *t*_*P*_ minutes, which ensures that foragers do not resume foraging until the predator is out of detection range.

*Grouping:* For grouping, foragers adjust their MOVE direction according to the position of neighbors (S2 Video). We use grouping rules from a previous study [45], based on repulsion-alignment-attraction models [46–49], which represents a simple mechanism that has been shown (in models) to generate various grouping patterns through self-organization. The rules are: (i) If *n*_*RZ*_ > *n*_*R*_, foragers turn away from neighbors in a repulsion zone *z*_*R*_, where *n*_*R*_ defines how many neighbors a forager tolerates in its repulsion zone, and *n*_*RZ*_ is the actual number of neighbors in its repulsion zone; (ii) If *n*_*RZ*_ ≤ *n*_*R*_, foragers turn towards neighbors in an attraction zone *z*_*A*_ (where *z*_*R*_ ≤ *z*_*A*_ ≤ 50 m), and towards the average direction of foragers in an alignment zone *z*_*L*_ (where *z*_*R*_ ≤ *z*_*L*_ ≤ 25 m). The angle that foragers turn is determined by rotating a forager’s heading towards a preferred heading, which depends on whether the forager is repulsed or attracted. To allow for variation in responsiveness to neighbors, we include maximal turning angles: a repulsion turning angle (*a*_*R*_) and an attraction turning angle (*a*_*A*_). If the angle between a forager’s heading and the preferred heading is more than the maximal turning angle, then the forager only turns up to the maximal turning angle.

**Life-history:** Foragers reproduce when their energy reaches a threshold (10^5^). Energy increases when the energy intake from food (2 energy units per item) exceeds metabolism (1 energy unit per minute). The offspring inherits half the parent’s energy, and evolvable parameters (Table 1). Evolvable parameters mutate with probability 0.05, in which case the offspring’s parameter value is drawn from a normal distribution centered on the parent’s parameter value, with a standard deviation scaled relative to values expected for that parameter (Table 1). The mutation rate was chosen operationally, such that grouping was observed to evolve. Foragers live maximally 20 years, covering multiple reproductive events, but generally die earlier due to starvation, a stochastic death rate, and predation.

**Environment:**

*Resources:* Food is uniformly distributed in the environment, at a density that supports the forager population (0.535 resource units per m^2^). Food items disappear when eaten, and regrow over time such that on average there is a regular renewal of resources. Since resources are depleted locally through foraging, foragers need to move to find food. In addition, foraging competition is generated within groups.

*Predators:* the two predators are solitary, and do not die or reproduce. A predator moves forward continually, until a forager is within 50 meters. The closest forager is then approached up to a critical distance *d*_*P*_, at which point the predator attacks and catches the forager. The predator can immediately hunt again.

For further model details please see Supporting Information S1 Text.

## Analysis

We ran evolutionary simulations with three levels of predation risk. Predation risk was varied by changing a critical distance (*d*_*P*_ = 5, 7, 9) that the predator had to approach in order to catch a forager. Evolutionary simulations where initialized with vigilance and foraging parameters that had evolved in solitary populations (see Supporting Information S2 Text).

To obtain an overview of evolved parameters, we conducted ancestor traces on evolutionary simulations, starting with the final population and backtracing to the beginning. Average parameters from ancestors at the end of the simulation (between year 800–900) over all simulations were taken as evolved parameters. For group size (number of neighbors within 50 meters), we calculated averages over the last 50 simulation years.

More detailed lineage analysis was conducted by repeating ancestor traces from different time points (e.g. at 20 year intervals) and merging them. This reveals lineages that go extinct before the end of the simulations, which can represent interesting events or dynamics, such as periods with co-existing genotypes. Such genotypes and the effect of their interactions on fitness were studied using different kinds of simulation: (i) invasion simulations where one or more genotypes could invade a population of one or more other genotypes, i.e. one genotype changes into another via a single mutation, but no other mutational changes occur; (ii) controlled evolutionary simulations where (a) vigilance was fixed, or (b) vigilance could only mutate between two values, but grouping parameters could evolve freely.

In the following, we focus on the vigilance and grouping parameters that are necessary to explain our results. For a complete overview please see Supporting Information Texts S2 and S3.

## Results

**Figure 2:**
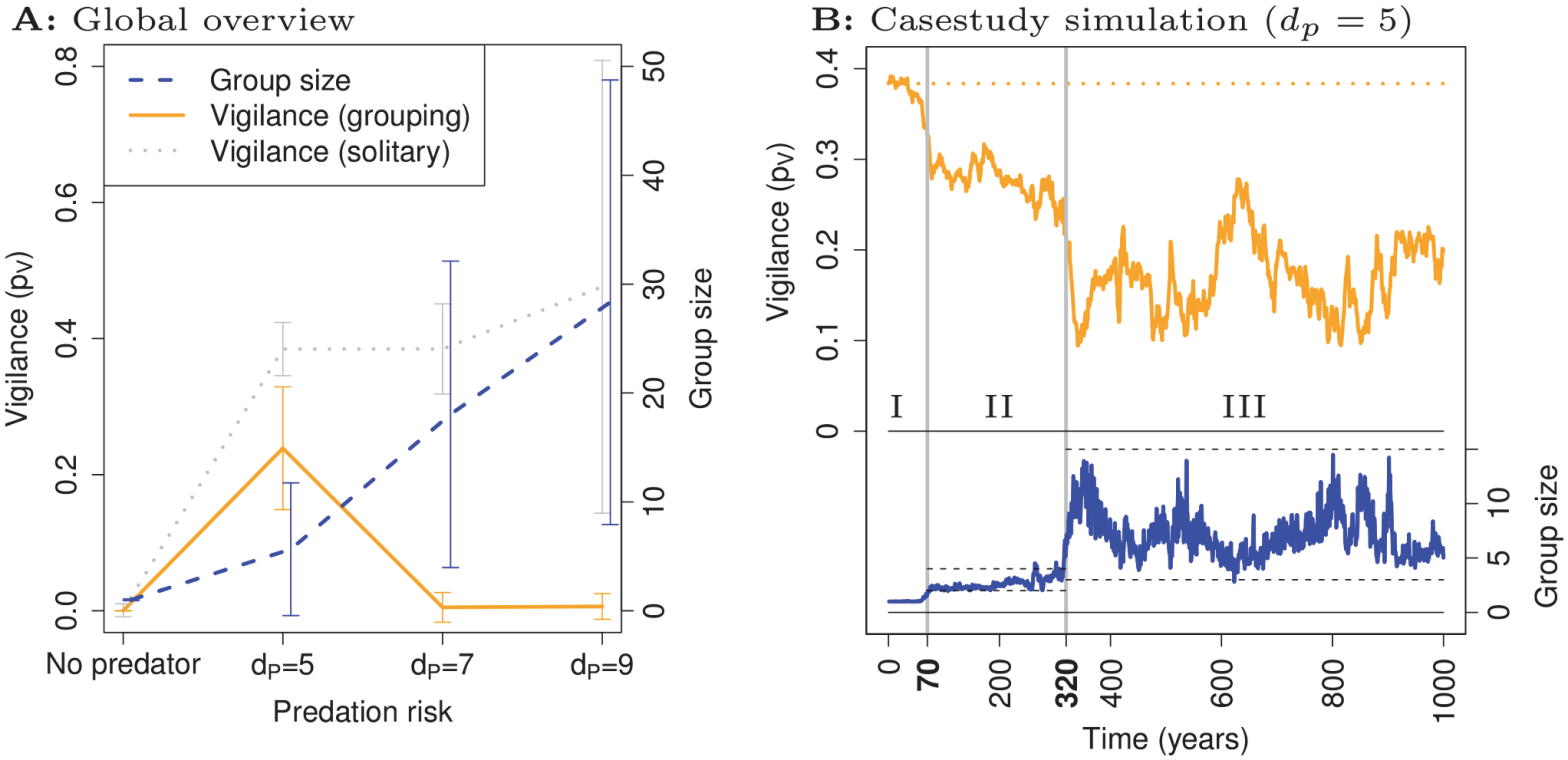
Overview and case-study. A: An overview of evolutionary simulations reveals that larger groups evolve when predation risk *d*_*P*_ is greater (blue dashed). Compared to simulations without evolution of grouping (grey dotted), vigilance evolves to smaller values (orange solid) if grouping evolves. Shown are means and standard deviation. B: The time course of mean group size (blue) and mean vigilance (orange) in a case-study for *d*_*P*_ = 5 exhibits three phases: (I) a solitary phase; (II) a small group phase; and (III) a large group phase where group size and vigilance co-evolve: when group size increases vigilance declines, and vice versa.

We find that grouping evolves in response to predation, and larger groups evolve when we increase the distance *d*_*P*_ at which predators catch foragers (Fig. 2A, blue): grouping dilutes the risk of being caught per predator attack, and contagious fleeing spreads information about predators (see Supporting Information S4 Text). Vigilance (*p*_*V*_) evolves to smaller values in groups (orange) than in populations of solitary foragers (grey), and vigilance completely disappears under high predation risk when groups are large (*d*_*P*_ = 7 and *d*_*P*_ = 9, Fig. 2A).

To study its co-evolution with grouping, we focus on the case where vigilance is present (*d*_*P*_ = 5). Typical simulations exhibit three evolutionary phases: (I) a solitary phase; (II) a small group phase (mainly pairs); and (III) a large group phase with fluctuations in group size (Fig. 2B, blue). Of 10 simulations, 9 reached phase II, and 7 of those reached phase III. In phase III a co-evolutionary dynamics between group size and vigilance can be observed, where vigilance (Fig. 2B, orange) decreases when larger groups (Fig. 2B, blue) evolve, and vice versa. To understand this evolutionary trajectory (Fig. 2B), we determined what types of individual were present in phase II and III, and studied their behavior.

**Figure 3:**
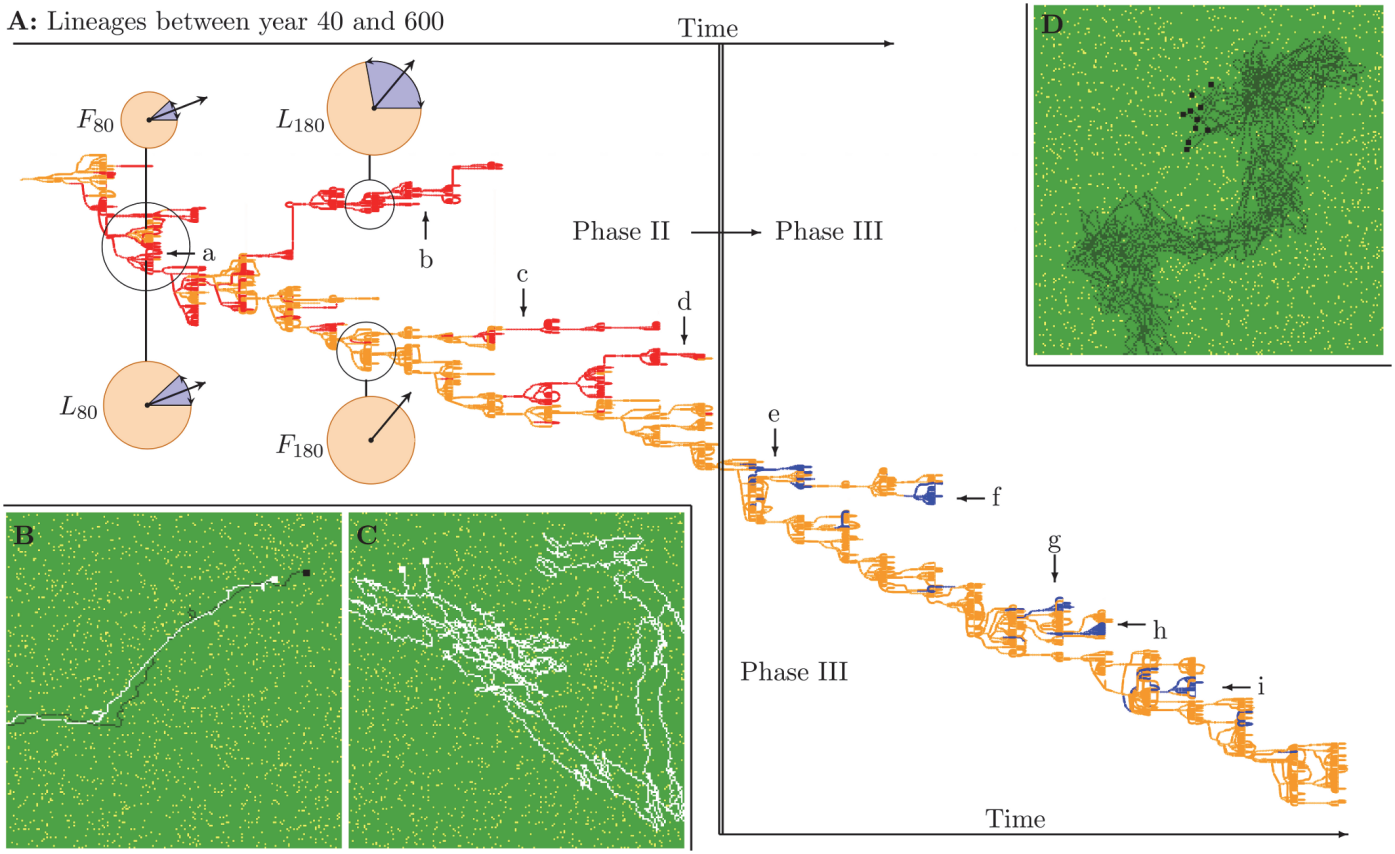
Lineages and behavior in phase II and III. A: Lineages between year 40 (top left) and 600 (bottom right), with the transition from phase II to III (crossing thick vertical line, year 320). At the origin of grouping (circle at a), there are two co-existing lineages (red and orange) which differ in their repulsion zone (radius of orange circle *F*_80_ and *L*_80_, where the blue segment shows the repulsion turning angle *a*_*R*_, and the large arrow indicates the forager’s heading). This difference in repulsion zone leads to the leader-follower movement shown in B, which is needed to overcome the lack of coordination when two leaders (or followers) form groups (shown in C). Later, leaders and followers are differentiated in the repulsion turning angle (*F*_180_ and *L*_180_). Red lineages b, c, and d are independently evolved leader lineages which co-exist with followers. During the transition from phase II to III, followers become independent from leaders and bigger groups evolve (shown in D). From this point on, non-vigilant lineages (*p*_*V*_ = 0, blue) repeatedly evolve and go extinct (small arrows e to i). B–D: Visualization of movement of leader-follower (B) and two followers (C) at year 70, and movement of group foragers at year 400 in phase III (D).

**Phase II: Leaders and followers.** After the origin of grouping (at the start of phase II), foragers have weak grouping tendencies and form pairs. Lineage analysis over the period from year 40 to 320 reveals multiple co-existing lineages during phase II (Fig. 3A, red (arrows a to d) and orange lineages), even at the origin of grouping (Fig. 3A, circle at lineage a). Foragers in orange lineages are more strongly repulsed by neighbors than those in red lineages (see below). This difference in tolerance leads to the formation of “leader-follower” pairs (Fig. 3B and S3 Video).

The difference in tolerance evolves via a differentiation in the repulsion turning angle *a*_*R*_, and/or the repulsion zone *z*_*R*_. Initially (year 80) leaders and followers are differentiated in their repulsion zone *z*_*R*_: 21.06 in leaders and 13.74 in followers, with a repulsion turning angle *a*_*R*_ of 0.373 (Fig. 3A, *F*_80_ and *L*_80_). Later (year 140–230) these differences are reduced (*z*_*R*_ is 21.06 in followers and 22.5 in leaders), and instead the two genotypes differ in the repulsion turning angle (*a*_*R*_ is 0.0 in followers and 0.825 in leaders; Fig. 3A, *F*_180_ and *L*_180_). Both types of differentiation generate leader-follower movement.

The difference in tolerance leads to leader-follower movement because the intolerant type moves away and thus “leads” the tolerant type, which ends up “following” (S3 Video). In contrast, leader-leader or follower-follower pairs (Fig. 3C and S4 Video), exhibit parallel movement with frequent changes in direction (Fig. 3C and S4 Video), while larger groups of one type form rigid immobile structures (S7 Video). This happens because foragers in leader-leader, or follower-follower pairs, have the exact same repulsion and attraction tendencies: foragers move to the position at which attraction and repulsion are balanced, causing rigid spacing. Such groups therefore remain longer in areas depleted of food. In contrast, the leader-follower differentiation generates coordinated travel, and keeps groups small: the position of the follower effectively represents a memory of where the pair just came from, enabling the pair to move away from areas depleted of food. Thus they forage efficiently. Moreover, larger groups with both leaders and followers tend to split up into a number of mixed pairs (S6 Video).

Invasion simulations reveal that only in mixed pairs, leaders and followers can invade and replace solitary foragers (results available on request): leaders and followers are therefore mutually dependent via a “traveling mutualism”. This mutual dependency constrains the evolution of larger groups (see Supporting Information S5 Text), causing evolutionary stasis in group size (Fig. 2B, phase II).

The constraints on group size are released when followers evolve independence. Independence becomes possible after followers evolve very small, or no, repulsion turning angle (see Supporting Information S6 Text), after which the repulsion zone can be thought of as a “tolerance zone”: foragers either are attracted to neighbors, or ignore them. This enables flexible movement when neighbors are within the repulsion zone. In addition, over time (year 100–320) the attraction turning angle *a*_*A*_ declines for both followers and leaders (see Supporting Information S6 Text). A smaller attraction turning angle limits the maximal turn of foragers, reducing reactivity to neighbors. An overall group heading is therefore more easily maintained: followers at year 140 and at year 250 split into sub-groups more readily, and move more effectively, when initialized in large groups with their own kind (S8 Video), than leaders (S7 Video). Since a large repulsion turning angle is a pre-requisite for being a leader, leaders cannot form efficient groups of their own kind, and they go extinct when followers become independent. Followers therefore inherit the world, generating the transition to phase III.

**Phase III: Eco-evolutionary dynamics of group size and vigilance.** In phase III larger groups (S9 Video) and reduced vigilance evolve after the constraints on group size in phase II have been released (Fig. 2B, from phase II to III). However, group sizes remain relatively small compared to simulations with a greater risk of predation (Fig. 2A, compare *d*_*P*_ = 5 to *d*_*P*_ ≥ 5). Thus, while there is a trend towards larger groups and less vigilance after phase II (Fig. 2B, from phase II to III), at some point less vigilant foragers with stronger grouping tendencies can no longer replace the existing population: there is a reversal of selection and new constraints on group size arise, and relatively small groups with vigilance are maintained.

**Figure 4:**
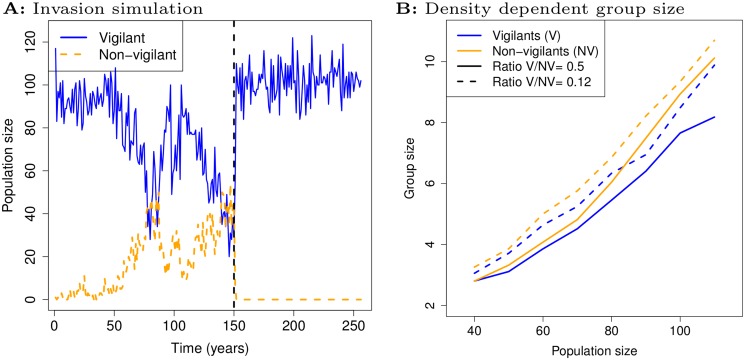
Co-existence of vigilant and non-vigilant foragers. A: Invasion simulation showing a selected non-vigilant genotype (orange) invading a population of a selected vigilant genotype (blue), after which both types co-exist. The non-vigilant genotype has a stronger grouping tendency and this enables it to coexist with the vigilant genotype. Co-existence ends when non-vigilant foragers are prevented from fleeing when vigilant foragers flee (after dashed line). Parameters for vigilant and non-vigilant foragers were taken from year 330 in the simulation of Fig. 2B. B: Average group size in vigilant (blue) and non-vigilant foragers (orange), using the same genotypes as in A, decline as population size declines. We used simulations in which population size and the ratio of vigilant and non-vigilant foragers was fixed. A greater proportion of non-vigilant foragers (dashed lines) leads to larger groups sizes since non-vigilant foragers have stronger grouping tendencies.

Lineage analysis reveals that non-vigilant foragers repeatedly evolve and go extinct (Fig. 3A, blue lineages indicated by short arrows e to i). In simulations where we stop mutations, we find that vigilant and non-vigilant foragers can co-exist, but only when non-vigilant foragers can use the information about predators provided by vigilant foragers (Fig. 4A). The non-vigilant foragers are therefore dependent on the vigilance of others: non-vigilant foragers are too unproductive to compete with vigilant foragers when information transmission is prevented. Relative to the mutualistic leader-follower societies of phase II, the population has therefore evolved to a socio-ecological setting characterized by a population of vigilant foragers in relatively large groups, which (at least periodically) hosts non-vigilant information-parasites.

Non-vigilant foragers have a foraging advantage within groups, since the time they do not use for vigilance is used for foraging. However, non-vigilant foragers can only invade vigilant populations when large enough groups have evolved (see Supporting Information S7 Text), and even then, non-vigilant foragers require stronger than average grouping tendencies to specialize on the largest groups in the population. This is because the parasitization by non-vigilant foragers is not perfect: Vigilant foragers always have a survival advantage [18], because they always benefit from their own vigilance (see Supporting Information S7 Text). The survival advantage is greatest in small groups and is negatively frequency-dependent: The survival advantage increases when the proportion of vigilant foragers declines (see Supporting Information S9 Text). As a result, the invasion of non-vigilant foragers has several ecological repercussions: (i) overall vigilance is reduced and death rates increase, which (ii) reduce both the population size (see Supporting Information S7 Text) and global competition for resources; and as a result (iii) group sizes decline, because grouping depends on population density (Fig. 4B). All these factors cause the survival advantage of vigilant foragers to increase, so that eventually non-vigilant foragers lose their fitness advantage (see Supporting Information S7 Text). In this way, non-vigilant and vigilant genotypes with fixed grouping tendencies can (in principle) co-exist. This negative frequency-dependence and the dependency of group size on population density define the ecological context in which natural selection operates in phase III.

**Figure 5:**
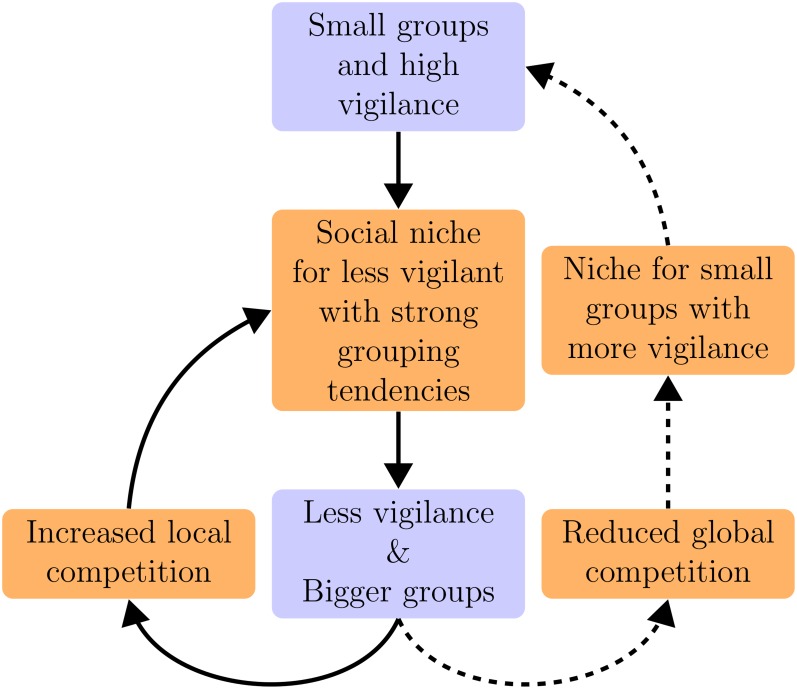
Eco-evolutionary interactions of vigilance and group size in phase III. Relatively small groups with high vigilance evolve after phase II, but this generates a niche for less vigilant foragers. Reduced vigilance and larger groups therefore evolve, which reinforces the social niche of less vigilant foragers. This can generate a within-group selection cycle (solid arrows). However, non-vigilant foragers in large groups forage inefficiently, leading to a reduced global competition for resources. As a result the group-level selection of ‘small group’ foragers with high vigilance becomes possible, and these can outcompete large groups with less vigilance. The population can therefore return to the situation with small groups and high vigilance (dashed arrows), where the niche for less vigilant foragers will re-emerge. Evolutionary changes are in blue and there ecological consequences are in orange.

We used controlled evolutionary simulations to study the effect of evolving grouping tendencies (see Supporting Information S8 Text). These simulations start with vigilance fixed at *p*_*V*_ = 0.15 (first 50 years), allowing only grouping parameters to evolve, to establish the evolutionary attractor for group size for this vigilance level (optimized group size). After year 50, we allow vigilance to mutate between vigilance (*p*_*V*_ = 0.15) and non-vigilance (*p*_*V*_ = 0.0), and non-vigilant foragers readily invade the population (see Supporting Information S8 Text). Initially larger groups evolve, probably to compensate for increasing death rates. As a result, a within-group selection feedback is generated, where less vigilance leads to larger groups and increased within-group competition, which in turn leads to less vigilance (Fig. 5, solid lines).

The within-group selection mainly affects large groups, because small groups are less, or not, affected by non-vigilant parasites. Therefore, although ‘small group’ vigilant foragers with weak grouping tendencies were outcompeted by ‘optimized group’ vigilant foragers in the absence of parasites, the invasion of non-vigilant parasites causes ‘optimized group’ vigilant foragers to lose their competitive edge. This means that a niche for ‘smaller than optimized’ group sizes emerges again (Fig. 5, dashed lines), and new ‘small group’ vigilant lineages can evolve (see Supporting Information S8 Text).

Such group size evolution can only occur if different grouping tendencies indeed result in different lifetime group sizes, which is evident from the difference in group size between vigilant and non-vigilant genotypes, where the latter has stronger grouping tendencies (Fig. 4B, compare blue to orange). This group-size differentiation is the aggregate outcome of an assortment process in groups that emerges without explicit grouping preferences (see Supporting Information S9 Text): Foragers with weak grouping tendencies end up on the periphery of groups, and are more likely to split off together into small sub-groups (S10 Video). As a result, group size can be selected for on a group-level, and small groups can take over the population. However, even if non-vigilant foragers go extinct, they eventually return because the vigilant population evolves to optimized group sizes again (see Supporting Information S8 Text), where the niche of non-vigilant parasites re-emerges (Fig. 5, going from dashed lines back to solid lines). The direction of selection for group size and vigilance is therefore determined by group sizes in the population, and how the productivity of different group sizes is affected by parasitization. This pattern of co-evolving group size and vigilance (Fig. 2B) offers an explanation for the repeated emergence and extinction of non-vigilant lineages (Fig. 3A).

We therefore ran evolutionary simulations where we doubled the population (i.e. double the area and carrying capacity of the environment), and reduced the mutation rate (from 0.05 to 0.0125). We find that group size fluctuations are dampened when both the population size is doubled and the mutation rate is dropped (see Supporting Information S11 Text). The possibility of reduced amplitude of fluctuations indicates that stochasticity and evolutionary drift could amplify the dynamics, or that the dynamics depend on the relative rates of evolutionary and ecological processes. To distinguish between these and other alternatives deserves further study, but is beyond the scope of this study. Important for the present study is that for the population sizes and mutation rates we studied, we observe relatively small groups with vigilance being maintained. This maintenance can only occur if the invasion of less vigilant foragers with stronger grouping tendencies is halted, due to the fact that groups with vigilance are more productive than non-vigilant ones, and the small groups that limit parasitism can indeed be selected for.

Thus, when predation risk is greater (*d*_*P*_ ≥ 7) the maintenance of vigilance does not occur, because the size of optimized groups shifts to larger values (see Supporting Information S10 Text). In this case, vigilant foragers can no longer compete with non-vigilant foragers, because the small group sizes that the vigilant foragers need for escaping parasitism, are now too small for appropriate survival and foraging. The result is large groups with no vigilance (Fig. 2A, *d*_*P*_ ≥ 7 and S11 Video).

## Discussion

To determine how social evolution is affected by the specifics of individuals and eco-evolutionary feedback, we examined a simplistic evolutionary case-study, focusing on the co-evolution between grouping, vigilance and foraging. Our results reveal an evolutionary trajectory with multiple evolutionary phases with different forms of sociality, namely (i) societies with leader-follower pairs, and (ii) small-group societies with fission-fusion dynamics. There are three main implications of our results that are relevant for understanding the evolution of sociality.

First, the kinds of society that evolve in our model depends on the way that individual-level details affect social patterns and their inter-relation: i.e. particular details of individuals can determine evolutionary outcomes and dynamics. Thus, the leader-follower societies (Fig. 2B, phase II) evolve because of constraints on individual cognition with respect to coordinated movement in groups. This happens because the foragers in the model cannot remember a general travel direction, and are either attracted to and aligning with, or repulsed from neighbors. As a result, coordinated travel is difficult, even though it is essential for foraging efficiently. Evolution therefore “comes up with” an ingenious solution: a leader-follower mutualism that generates coordinated travel despite the cognitive constraints of the foragers. Leader-follower travel enables directed travel away from areas depleted of food (S3 Video), and affects evolutionary dynamics by generating evolutionary constraints on group size. More trivially, the evolution of fission-fusion societies in phase III (Fig. 2B, phase III) depends on constraints on social structure: there is no direct way for foragers to form relationships and group preferentially with particular foragers. Permanent bonded groups therefore cannot evolve, although such social structure could affect how multi-level selection operates.

Second, our results support theory demonstrating that the co-evolution of grouping with cooperative social traits enables the evolutionary maintenance of small groups where cooperation can thrive [36]. In our model, cooperation is not predefined, but emerges from synergy generated by grouping. This cooperation is maintained via the co-evolution of social traits in two qualitatively distinct ways. On the one hand, a seemingly unrelated social trait, namely coordinated travel, indirectly helps maintain cooperative vigilance in leader-follower societies: For long periods of time, the necessity for coordinated travel prevents the evolution of group sizes where a cooperative dilemma arises. On the other hand, in phase III, like in the model of [36], there is a cooperative dilemma. In phase III, non-vigilant foragers evolve, and these are analogous to freeloaders: Within groups, non-vigilant foragers benefit from, but do not contribute to, a public good provided by vigilant foragers. Like [36], we find that freeloaders reduce group productivity and have stronger grouping tendencies than cooperative individuals. As a result, cooperation is maintained directly via group-level selection for relatively small groups with greater levels of cooperation.

Important in our model, is that the relative fitness of foragers with different vigilance levels depends on group size. Thus, non-vigilant foragers can no longer benefit from freeloading when groups are too small. As group sizes decrease, the socio-ecological context therefore changes from a cooperative dilemma with freeloaders, to a mutualistic one where freeloading is not advantageous. Small groups therefore provide a ‘refuge from freeloading’. Thus there is group-level selection for group sizes where freeloading is reduced, made possible by assortment into different sized groups via differences in grouping tendencies.

However, in Avilés’ case, the social context is by definition one where freeloading is possible, unless individuals are solitary. Thus, Avilés’ model concerns an idealized hard-case scenario, and she provides a proof of principle that the co-evolution of group size and cooperation can maintain cooperation without a ‘refuge from freeloading’. It is possible that this result depends on individuals spending their lives in a single group, as is assumed in her model. Thus, it remains to be seen whether for fission-fusion societies, as in our model, a ‘refuge from freeloading’ is a prerequisite for the maintenance of small groups with vigilance. The loss of vigilance when predation risk increases (Fig. 2A, *d*_*P*_ > 5), suggests that such a ‘refuge’ is important in our model. Our results therefore highlight that emergent social processes may not exactly fit idealized hard-case scenarios, and that group-level selection can operate in fission-fusion societies due to assortment via grouping tendencies.

The third implication is that by including specific individual details, and not predefining costs and benefits, we can study how eco-evolutionary feedback generates evolutionary trajectories that progress through several phases with different forms of sociality. In game-theoretic terms this is equivalent to understanding how particular games originate and evolve into other games. At present, evolutionary game theory generally does not address how games (strategies and pay-off matrices) originate and evolve.

Our study shows that the specifics of individuals determine which ‘games’ arise, as well as how they evolve into different ‘games’: Cognitive constraints lead to leader-follower travel as a means to achieve coordinated travel, which in turn generates constraints on group size evolution, and hence a distinct socio-ecological attractor, namely mutualistic leader-follower societies. This is analogous to a type of coordination game, or rowing game [50], where group-level selection dominates. The same mechanistic details determine that constraints on coordinated travel are overcome, and a new socio-ecological attractor evolves, namely fission-fusion societies with a cooperative dilemma. This could be represented as a complex public goods game [51], with a prominent role for within-group selection. In this way, we show how it is possible to generate theory about how particular socio-ecological situations (‘games’) evolve and change via eco-evolutionary feedback, and how such evolutionary changes relate to mechanistic details and multi-level selection.

Relating our results to the evolution of sociality in real animals, such as primates and antelope, requires taking simplifying assumptions into account. For instance, many animals are phenotypically flexible and behaviorally sophisticated, and do not have grouping and vigilance levels that are fixed over their lifetime, unlike the foragers in our model. Thus the time-scale on which group size and vigilance vary, is expected to be shorter in real animals.

More specifically, we expect two ways in which changes in behavioral sophistication could affect the evolution of leader-follower societies: (i) Greater behavioral flexibility would allow leader and follower types to be generated phenotypically, rather than genetically: In non-evolutionary models it has been shown that leader-follower movement can self-organize from differences in dominance [52] or differences in energy reserves [53]; (ii) Greater cognitive sophistication, such as memory of a general travel direction, may cause the difficulties of coordinated travel to disappear completely, in which case leader-follower societies would not evolve. Our results therefore suggest that leader-follower travel may be a pre-requisite for coordinated travel if there are cognitive constraints, and that leader-follower patterns can emerge easily from an asymmetry in motivation to move. For instance, in various bird (e.g. ducks) and mammal (e.g. antelope) species, offspring follow their mothers. Such patterns may simply be the result of offspring having a greater motivation to follow their mother, than vice versa.

In our model we also assume a strong trade-off between vigilance and foraging, and find that vigilance can only be maintained in relatively small groups. However, vigilance is present in various animal species in much larger groups, and in primates, vigilance does not clearly decline with increasing group size [54]. This implies that the trade-off is less strong in various natural settings, for instance because individuals are also vigilant for other reasons, such as keeping track of group members. Our results therefore provide a baseline for studying how phenotypic flexibility affects the evolutionary dynamics of group size and vigilance, and for studying how additional social complexity can affect the vigilance-foraging trade-off.

Given these simplifications, directly comparing the specific evolutionary trajectory that arises in our model to that of particular animal species would be misplaced. Instead, our results emphasize how evolutionary case-studies can be used to study the intermediate stages that are involved during the evolution of particular forms of sociality. Intermediary stages reveal how selection pressures change as societies evolve. As a result, the distinction between ‘how societies evolve’ and the ‘present day function of sociality’ comes into focus. This distinction is necessary for understanding shifts in functionality of traits, for example the shift in function of grooming from primarily hygienic purposes to playing a major role in social bonding in primates. In fact, this shift may be part of an evolutionary feedback between cognitive abilities and social bonding, which has been proposed to have driven the evolution of large brains and complex societies in the primate lineage [4, 9]. If true, it remains to be seen how such evolution depends on intermediate stages and the particular details of primates, as opposed to those of antelope, where such an evolutionary feedback apparently did not arise. Studying such issues requires elaborate evolutionary case-studies, and a start can be made by extending our model with other kinds of social interactions, such as grooming, and more sophisticated cognitive processing. In this way, we envisage that the dynamics of the co-evolution of grouping with a multitude of individual characteristics can be unraveled, so that we can better understand how different forms of sociality may have evolved.

## Supporting Information

S1 Text Detailed model description. (PDF)

S2 Text Evolved vigilance and foraging parameters. (PDF)

S3 Text Evolved grouping parameters. (PDF)

S4 Text Dilution of risk and contagious fleeing. (PDF)

S5 Text Constraints on group size evolution. (PDF)

S6 Text Evolution of repulsion and attraction in leaders and followers. (PDF)

S7 Text Co-existence of vigilant and non-vigilant foragers and the survival advantage of vigilant foragers. (PDF)

S8 Text Controlled evolutionary simulation. (PDF)

S9 Text Assortment and grouping tendencies. (PDF)

S10 Text Optimal group size and increased predation. (PDF)

S11 Text Population size and mutation rate. (PDF)

S1 Video Predation on solitary forager. Solitary individual foraging on food items. Large white square: predator; Small black square: forager; Around forager: dark green flashing area illustrates how it scans for the predator. Green: background; Lighter green circles: food items in patches; This represents about 250 minutes of foraging viewed on a 400 by 400 meter area. On the first approach the predator is detected and the forager flees (turns white). It then resumes foraging, and is caught by the predator on the second approach.
(MP4)

S2 Video Predation and grouping. A group of foragers (black squares) scanning for food. A predator (large white square) approaches, but the foragers flee (turn white) when the predator is detected: the detecting forager flees and triggers all its group mates to flee. Scanning for food stops and all foragers wait “up a tree” until the predator has departed. Once all the foragers are safe, the predator ignores them and looks for new targets. In this case none are in view and it continues to move forward. Dark green is empty space, lighter green circles are food items.
(MP4)

S3 Video Leader-follower pair from year 70. A leader-follower pair foraging from year 70 in Fig. 2A. White with light green trail: FOLLOWER; Black with dark green trail: LEADER; Green: background; Lighter green spots: food items; This represents 200 minutes of foraging on a 400 by 400 meter area.
(MP4)

S4 Video 2 followers from year 70. A pair of two followers from year 70 in Fig. 2A, both white with light green trail. Green: background; Lighter green spots: food items; This represents 200 minutes of foraging on a 400 by 400 meter area. Similar results are obtained with 2 leaders.
(MP4)

S5 Video 10 leaders from year 70. 10 leaders foraging from year 70 in Fig. 2A: black with dark green trails. Green: background; Lighter green spots: food items; This represents 200 minutes of foraging on a 400 by 400 meter area. Similar results are obtained with 10 followers.
(MP4)

S6 Video 7 leaders and 3 followers from year 70. 7 leaders (black with dark green trails) and 3 followers (white with light green trails) from year 70 in Fig. 2A. Green: background; Lighter green spots: food items; This represents 400 minutes of foraging on a 400 by 400 meter area.
(MP4)

S7 Video 10 leaders from year 140. 10 leaders from year 140 in Fig. 2A: white with light green trails. Green: background; Lighter green spots: food items; This represents 300 minutes of foraging on a 400 by 400 meter area.
(MP4)

S8 Video 10 followers from year 140. 10 followers from year 140 Fig. 2A: black with dark green trails. Green: background; Lighter green spots: food items; This represents 300 minutes of foraging on a 400 by 400 meter area.
(MP4)

S9 Video 10 group foragers from year 400. 10 foragers from year 400 in Fig. 2A: black with dark green trails. Green: background; Lighter green spots: food items; This represents 300 minutes of foraging on a 400 by 400 meter area.
(MP4)

S10 Video Assortment. 20 foragers from year 189 in Fig. 1 of S8 Text: black foragers are those with vigilance (*pV* = 0.15) and weak grouping tendencies; white foragers are those with no vigilance (*pV* = 0.0) and strong grouping tendencies. Green: background; Lighter green spots: food items.
(MP4)

S11 Video Large group. 50 foragers from a uniform environment with high predation pressure (dP = 9): black with dark green trails; White: focal individual. Green: background; Lighter green spots: food items; This represents 200 minutes of foraging on a 400 by 400 meter area.
(MP4)
