# Supplementary material for: The Evolution of Different Forms of Sociality: Behavioral Mechanisms and Eco-Evolutionary Feedback
Source: PLoS One. 2015 Jan 28;10(1):e0117027. doi: 10.1371/journal.pone.0117027 (PMC4309640; doi:10.1371/journal.pone.0117027)
Supplement: S1 Text — (PDF) [file pone.0117027.s001.pdf]

Text S1: Detailed model description

Daniel J. van der Post[1,2,3,*], Rineke Verbrugge[1], Charlotte K. Hemelrijk[2]
1 Institute of Artificial Intelligence, University of Groningen, P. O. Box 407, 9700 AK, Groningen, The Netherlands
2 Behavioural Ecology and Self-Organization, University of Groningen, P. O. Box 11103, 9700 CC Groningen, The Netherlands
3 Centre for Social Learning and Cognitive Evolution, School of Biology, University of St. Andrews, Queens Terrace, St. Andrews, Fife KY16 9TS, United Kingdom
∗ E-mail: d.j.vanderpost@gmail.com

Contents

1 Text S1: Detailed model description . . . 1
1.1 Purpose . . . 1
1.2 State variables and scales . . . 1
1.2.1 Specification choices of scaling context . . . 3
1.3 Process overview and scheduling . . . 5
1.4 Design concepts . . . 5
1.5 Initialization . . . 6
1.6 Input . . . 6
1.7 Submodels . . . 6
1.7.1 Forager continuous updates and decision making algorithm . . . 6
1.7.2 Behavior actions of foragers . . . 7
1.7.3 Forager discrete updates . . . 9
1.7.4 Predator decision making . . . 10
1.7.5 Predator actions . . . 10
1.7.6 Resources . . . 10

1 Text S1: Detailed model description

Here we document a full model description based on the ODD protocol for describing simulation models [1].

1.1 Purpose

The purpose of this model is to study the evolution of sociality due to predation. Specifically, we study how the evolution of individual-level interactions can generate novel social behavior through self-organizing processes. We address the role of self-organization in the evolution of social behavior and social complexity.

1.2 State variables and scales

Our model consists of a 2-dimensional environment with resources (Figure S1A), foragers and predators. Space and time are continuous, but some variables are updated via a discrete clock where each time point is a minute, a day is 720 minutes (only daylight), and a year is 365 days.

Resource items are described by: a spatial position, an energy value E_r, and a random time point in a year when they regrow if they are eaten. Items are placed singly with some probability per grid unit (0.535).

Foragers are characterized by the following state variables: age, energy, position, heading, behavior state, behavioral action, time to complete an action (completion time), a memory of the last foraging action, having a particular feeding target (i.e. a food item), an "internal clock" through which individuals

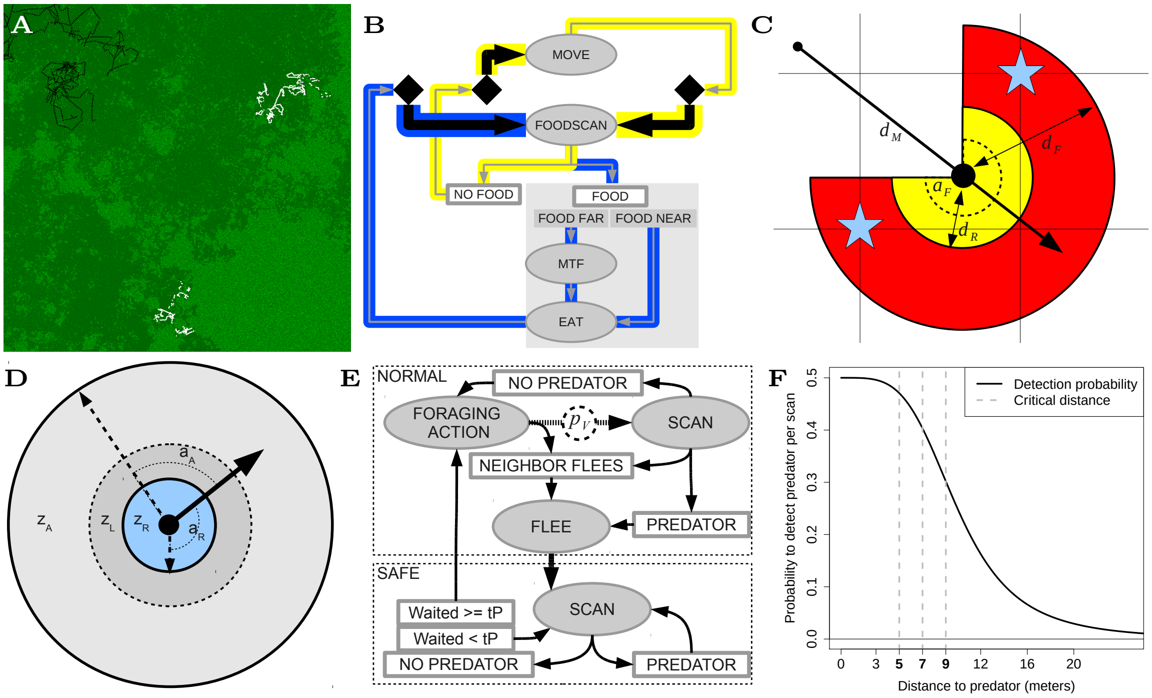

Figure S1. Model. A: An 800 by 800 meter section of the environment with food visible (light green; dark green is empty space), with trajectories of solitary foragers (black and white lines). B: Evolved decision-making algorithm for foraging actions from [2]: after MOVE, foragers do FOODSCAN. If foragers find food they EAT it, or first MOVETOFOOD (MFT). After EAT, foragers repeat FOODSCAN. If they do not find food, foragers MOVE forward. C: Visual representation of MOVE and FOODSCAN: d_M is the distance moved; the yellow zone is the foragers reach d_R; the red zone (including yellow zone) is the FOODSCAN area, given by radius d_F and angle a_F, where food items (blue stars) can be detected. D: Grouping rules: foragers are attracted to neighbors in the attraction zone z_A and align to them in the alignment zone z_L, with maximal turning angle a_A. Foragers are repulsed from neighbors in the repulsion zone z_R with maximal turning angle a_R. E: Decision-making algorithm for anti-predator actions: in between foraging actions (shown in B), individuals SCAN with probability p_V and continue foraging if there is no predator. If there is a predator, or if their neighbor flees, foragers flee and reach a SAFE state. Foragers continue to SCAN as long as they see the predator. After the predator has departed, foragers continue to SCAN for t_P minutes, after which they return to a NORMAL state and resume foraging. F: Predator detection function: the thick line shows how the probability to detect the predator declines with distance. Vertical dashed lines show the three critical distances d_P with which we ran evolutionary simulations.

time how long to wait in a safe position after fleeing from a predator, and “genes” coding for parameters that determine behavior (see Table 1, main text).

Predators are characterized by: position, heading, behavioral action, completion time and having a hunting target (i.e. a particular forager).

The forager population grows when individuals reproduce. Reproduction depends on energy intake via foraging. Foraging leads to depletion of resources, reducing resource density, food intake and birth rates. Carrying capacity is reached when birth rates slow down and equal death rates. At carrying capacity, competition for resource is maximal. Mutations in behavior “genes” during reproduction generates genetic

variation. If variation affects reproductive success, natural selection arises.

In some cases, initialized populations are not viable, because foragers are not vigilant enough, or cannot survive if there is no grouping. We therefore include a birth algorithm which maintains the population at a minimum of 10 foragers, where the forager with most energy has the greatest probability to reproduce: probability for individual i to reproduce $= (e_i/(\sum_{j=1}^{N} e_j))^n$, where e_i is an individuals energy level, N is the population size and $n(= 5)$ is the selection coefficient. Generally, the population grows beyond this stage, and this algorithm becomes redundant, but if not, we consider the population to be non-viable.

1.2.1 Specification choices of scaling context

The scaling context of our model is determined by fixed parameters (Table S1):

Spatial and temporal resolution: At the minimal level we define the minimal time and spatial scale. The minimal timescale is 1 second, which is the REACTIONTIME foragers have in order to FLEE after detecting a predator. Similarly the predator has REACTIONTIME in order to ATTACK if a forager flees. However, evolvable action durations can be minimally 10 seconds (t_{MIN}), to keep the model running fast enough. Space is continuous: individuals and resources can be placed at any given location. For convenience we place resource items on intersections of a 1 by 1 meter lattice.

Local information processing: Constraints on movement and perception (local information processing) are defined in terms of the spatial and temporal scale. Maximum speed is defined in terms of the minimal time interval, so that the duration of moving 1 meter can never be below t_{MIN}. The maximum range of resource detection (r_D) is then chosen to be small, so that individuals have to move to detect food. Within this range resource detectability is simply maximal, where an item of food can be found during a 10 second FOODSCAN in an area of 10 meters squared (Figure S1C yellow and red zone, i.e. a search angle of about 270 degrees with a radius of 2 meters). The individuals' REACH is then chosen to be below maximal resource detection range (Figure S1C yellow). This is quite a reasonable assumption (equal detection range and REACH is probably a limit case). The importance of this assumption is that individuals can MOVETOFOOD, which allows individuals to track contiguous stretches of food (i.e. patches) thus allowing "patch detection" on longer timescales (see [2]).

Maximum ranges for awareness (z_M, also for predators) and alignment (z_L) are chosen as follows (Figure S1D): we assume that for awareness (the position of a neighbor) both sight and sound can be used, giving 50 meters as a reasonable range, e.g for primates. However, this range is limited relative to the full size of the world so that individuals in different groups, or solitary individuals, are isolated and cannot interact, i.e. there should be space for different groups to arise. Moreover, for the alignment zone we assume a shorter range because alignment requires identifying the direction neighbors are moving. For identifying direction sound may also be used, but is probably more difficult than via visual cues. In addition we assume that predators can detect foragers as easily as foragers can detect each other, but that predators are less detectable because they are hunting, sneaking up, cryptic etc. Moreover, predator detection is such that predators have a reasonable chance to sneak up and reach the distance d_P where they can catch a forager before being detected.

The following relationships are therefore met:

$$d_R < r_D < d_P < z_L < z_M \tag{1}$$

Energy and life history: The minimal time interval sets a constraint on the minimal feeding interval t_{MIN_EAT}, namely SEARCH + EAT ($t_{MIN} + t_E$) and for simplicity we set $t_E = t_{MIN}$. t_{MIN_EAT} then sets the maximal energy accumulation rate in relation to energy per food item E_r and energy metabolism E_m: $(E_r/t_{MIN\_EAT}) - E_m$. Moreover, the energy per food item E_r in relation to energy metabolism E_m sets the maximum (average) feeding interval individuals can tolerate without net energy loss: $(E_r/t_{MAX\_EAT}) = E_m$ therefore $t_{MAX\_EAT} = E_r/E_m$. The energy required to reproduce $E_M/2$ defines the minimal birth interval t_{BIRTH}: $E_M/2((E_r/t_{MIN\_EAT}) - E_m)$. Thus the latter four parameters (t_{MIN}, E_r, E_m, E_M) define the minimal birth interval as well as minimal and maximal eat intervals.

Table S1. Non-evolvable parameters of forgers and environment.

Category	Parameter / description	Value	Units
Timescale	reaction time	1	sec
	t_{MIN} (minimal action duration)	10	sec
	day	720	min
	year	365	days
Environment	grid unit	1	m
	field size	5.66 x 5.66	km
Resources	renewal interval	1	year
	density	0.535	items per m^2
	detection distance	2	m
	detection probability	1	per sec per m^2
	t_E (handling time)	10	sec
	E_r (energy)	2	units
Individuals	d_R (individual reach)	0.9	m
	maximum speed	0.1	m/sec
	max neighbor awareness	50	m
	h (half max distance detect predator)	5	m
	N (scaling for predator detection)	5	
	E_m (metabolism)	1	units/min
	minimal energy	0	units
	E_M (maximum energy)	100000	units
	birth requirement	E_M	units
	birth energy costs	$E_M/2$	units
	offspring energy	$E_M/2$	units
	death rate	0.1	per year
	maximum age	20	years
	mutation rate	0.05	
Predator	d_P (predator attack distance)	5,7,9	m
	range for detecting foragers	50	m
	move distance	1	m
	move speed	0.1	m/sec
	attack speed	11.1	m/sec

By setting E_r, E_m and $E_M/2$ in relation to each other ($E_m < E_r <<< E_M/2$), we specify that: individuals must consume in the order of at least 10000 food items to reproduce, with a minimal birth interval of 14 days. This birth interval is quite low for mammals, but is never achieved because individuals have to move to find food and global depletion of resources lowers the foraging rate so that birth intervals increase to months-years. The reproductive cycle is therefore long enough to ensure that individuals can travel to and consume food from many locations and experience the full scale of patterns in the environment. Maximum life expectancy is roughly 10 years due to a death rate of 0.1 per year, chosen so that individuals can experience multiple reproductive cycles.

Here the relationships are:

$$t_E >= t_{MIN} \tag{2}$$

$$t_{MIN} + t_E < t_{MAX\_EAT} = E_r/E_m \tag{3}$$

$$E_m < E_r <<< E_M/2 \tag{4}$$

$$E_M/2((E_r/t_{MIN\_EAT}) - E_m)) = t_{BIRTH} >> t_{MAX\_EAT} \tag{5}$$

Environment: The environmental settings were chosen to support a viably evolving population at low enough densities to make grouping an issue (i.e. not just individuals aggregating by chance). Population size (carrying capacity) is defined in terms of the maximum feeding interval in relation to resource influx R_g into the environment: $t_{MAX\_EAT} * R_g$ (i.e. R_g divided by the minimum feeding rate). Thus, population size can be directly linked to the ratio $E_r/E_m (= t_{MAX\_EAT})$. The resource influx rate gives the total amount of resources per year R_T. The size of the world A then gives the max density of resources $R_d (= R_T/A)$.

Besides $A >> 0$, the following relationship is met to obtain the desired population size:

$$t_{MAX\_EAT} * R_g > 100 \tag{6}$$

1.3 Process overview and scheduling

We distinguish two levels of scheduling: (i) continuous time and (ii) discrete time steps (every minute).

Discrete updating: every minute we update individual age, reduce energy due to energy metabolism, check if an individual has enough energy to reproduce, determine if an individual dies due to a fixed death rate, starvation or old age. Also, all resource items with that minute as their regrowing time point, regrow if they had been eaten, and can then be detected and eaten by foragers.

Continuous updating: every behavioral action has a continuous time point at which it is completed (completion time). Actions can therefore end anywhere within a minute (with the resolution of "double" types in C programming language) and an action started in a previous minute is completed in the next minute. All individuals (foragers and predators) are put into a queue according to their completion time. The individual with the lowest completion time is next to complete its action and choose a new action. The completion time represents the duration of an action. Individuals therefore start their actions asynchronously, but action durations can overlap. This is an event-based scheduling. The sequence of actions of an individual is determined by its decision making algorithm (see Figure S1B and E).

Individuals can be activated by other individuals, for example when a neighbor flees. In that case, individuals do not complete their action, but interrupt it and choose a new action that is triggered by the activation (e.g. to also flee).

1.4 Design concepts

Emergence: our model is all about detecting and understanding emerging processes and the interactions between them. The following is a list of emerging phenomena:

- Foraging patterns: larger-scale detection of resource patterns beyond the local perception of individuals [2, 3].
- Grouping patterns: group size and group-level behavior [3].
- Patterns of resource depletion [3].
- Interactions between grouping and resource pattern detection [3].
- Interactions between predation and grouping: the object of study here.

Adaptation: there are at least two implemented levels of adaptation: (i) individuals have a decision making algorithm that determines how they respond to the local environment, and (ii) evolutionary adaptation of behavioral parameters via natural selection.

Fitness: we define that the rate of reproduction of foragers depends on energy intake, therefore more feeding generates a greater birth rate. A forager's lifespan depends on a fixed death rate, starvation, maximum age and how easily it is eaten by a predator. Fitness is therefore implicitly dependent on birthrate and lifespan and is not fixed. We study how fitness is affected by the evolution of foraging, anti-predator vigilance and grouping behavior.

Prediction: individuals do not predict future conditions, but respond to environmental conditions they encounter. These responses are embedded, in the sense that they are determined by locally observable conditions. We refer to this type of responsiveness as TODO, as in individuals DO WHAT THERE IS TO DO [4]. What individuals do is determined by evolvable decision making algorithms.

Sensing: central for TODO is local information processing, leading to larger scale TODO-based pattern recognition beyond the direct perception of the individual [2,5]. We only define local perception of food (2 meters), and on a somewhat larger scale the awareness of neighbors (up to 50 meters) and predators (up to 50 meters).

Interaction: foragers can approach, eat and deplete resources. Foragers can detect each other and approach, align or move away from other individuals. Predators can detect, approach, attack and eat foragers, and foragers can detect and flee from a predator.

Collectives: no collectives are pre-defined.

Observation: we collect data on the environment, foragers and predators. Environmental data can include state of depletion and patterns of depletion. For foragers data can include population size, spatial arrangement and grouping patterns, foraging patterns, lifespan, birthrates, predator detection rates, predator encounter rates. Analysis generally proceeds via macro-observables and becomes increasingly detailed as more information is needed to understand what processes are occurring (see main text).

1.5 Initialization

We initialize the environment with a fixed number of resource items, but different resource distributions. An initial population of 10 foragers is initialized with a given pre-evolved foraging genotype, and in some cases evolved anti-predator parameters. Individuals are initialized at random positions, start without an action, and with no information about the environment. Their first action will be FOODSCAN. Two predators are similarly placed in the environment.

1.6 Input

The main input variable is the intensity of predation. We consider uniform distributed resources and 3 intensities of predation specified via the distance at which a predator can attack and catch a forager: d_P.

1.7 Submodels

Here we present a "mathematical" skeleton of the model updating schedule:

1.7.1 Forager continuous updates and decision making algorithm

During each minute:
while any action ends this minute, take the individual with the soonest ending action:
(Note: predators are in the same scheduling list, so if soonest ending action is of a forager continue here, else move to section on predator behavior).
1) Complete Action

——* MOVE: get new position
——* FOODSCAN: get new food target or not
——* MOVETOFOOD: get new position
——* EAT: gain energy $e_i = e_i + E_r$
where e_i is individual energy and E_r is energy per food item
——* SCAN: detect predator or not
——* FLEE: individual changes to state SAFE + activates neighbors to FLEE
2) Choose New Action:
——* if (predator or neighbor flees) then FLEE (Figure S1E)
(Note that activation by neighbor or attacking predator cuts short
any active action, which is replaced by FLEE)
——* else (no predator)
———** if (RAND < p_V or if state == SAFE) then SCAN (Figure S1E)
———** else (Figure S1B)
————*** if (food target)
—————-**** if (distance to target < d_R) then EAT
—————-**** else MOVETOFOOD
————*** else (no food target)
—————-**** if (previous action = MOVE)
——————***** if (RAND < p_M) MOVE
——————***** else FOODSCAN
—————-**** if (previous action = EAT)
——————***** if (RAND < p_{SE}) FOODSCAN
——————***** else MOVE
—————-**** if (previous action = FOODSCAN)
——————***** if (RAND < p_{SN}) FOODSCAN
——————***** else MOVE
3) Get new completion time
4) Place forager in action cue sorted by completion time.

"Previous action" is always the last non-SCAN behavior, RAND is a random number between 0 and 1, p_V is the probability to SCAN for a predator, p_M the probability to repeat MOVE, p_{SE} and p_{SN} the probability to repeat FOODSCAN after EAT, or after not finding food, respectively, and d_R is the individual's reach.

1.7.2 Behavior actions of foragers

MOVE (Figure S1C, black line): get new heading

- Grouping (depending on grouping parameters, Figure S1D and Table 1):
  - if repulsion ($n_{RZ} >= n_R$) then $\vec{d} = -\frac{\sum_{j \neq i}^{n_{RZ}} \vec{r}_{ij}}{|\sum_{j \neq i}^{n_{RZ}} \vec{r}_{ij}|}$
    where n_{RZ} and n_R are the number of actual and tolerated neighbors in the repulsion zone z_R, respectively. $\vec{d_i}$ is the preferred direction of individual i, and $\vec{r}_{ij} = (\vec{p_j} - \vec{p_i})/|(\vec{p_j} - \vec{p_i})|$ is the unit vector in the direction of neighbor j, where $\vec{p_i}$ is the position of forager i.
  - else (attraction): $\vec{d_i} = \frac{\sum_{j \neq i}^{n_A} \vec{r}_{ij} + \sum_{j \neq i}^{n_L} \vec{v}_j}{|(\sum_{j \neq i}^{n_A} \vec{r}_{ij} + \sum_{j \neq i}^{n_L} \vec{v}_j)|}$
    where n_L is the number of foragers in the alignment zone z_L, n_A is the number of neighbors in the alignment z_L and attraction zone z_A, and $\vec{v_j}$ is the unit direction vector of neighbor j.
  - if (angle between $\vec{d}$ and $\vec{v} <= a_R$ or a_A) then $\vec{v} = \vec{d}$
    where a_R and a_A are the maximal turning angles for repulsion and attraction respectively.

- else turn a_R or a_A
  - $v_x = v_x cos(a) - v_y sin(a)$
  - $v_y = v_x sin(a) + v_y cos(a)$

- Random turn of a_M (if grouping happens, this follows grouping):
  - $t = a_M$
  - if (RAND < 0.5) t $= -a_M$
  - turn by angle t:
    - $v_x = v_x cos(t) - v_y sin(t)$
    - $v_y = v_x sin(t) + v_y cos(t)$

- Get new position and make sure it is on the grid:
  - $\vec{p'} = d_M \vec{v} + \vec{p}$
    where d_M is the MOVE distance, and $\vec{p'}$ and $\vec{p}$ are the new and old positions respectively.
  - while $\bar{(}p'_x \ or \ p'_y < 0$ or $p'_x \ or \ p'_y >= \sqrt{A})$
    where A is the area of the environment.
    - $\vec{v} =$ normalized random vector
    - $\vec{p'} = d_M \vec{v} + \vec{p}$

- Duration: $d_M * t_M$
  where t_M is the time it takes the forager to move 1 meter.

FOODSCAN (Figure S1C): try to get new food target

- for all n food items (distance $< d_F$ and angle $< a_F/2$ from heading)
  where d_F is the radius of search, and a_F is the angle about the foragers forward heading (as in Figure S1C).
  - per item: $p = \frac{t_F * 60}{\pi d_F^2 (a_F/\pi)}$
    where t_F is the duration of FOODSCAN.
  - if (RAND $< p$) then food item is detected
  - if new item is closer than previous choose new item

- Duration: t_F

MOVETOFOOD: get new position $d_R/2$ meters from food item

- $\vec{p'} = \vec{f} - \frac{d_R}{2} |\vec{f} - \vec{p}|$
  where $\vec{f}$ is the position of food item, d_R is the forager's reach and $\vec{p}$ the position of the forager

- Duration: distance moved * t_M

EAT: consume food item

- Duration: t_E

SCAN: detect predator or not (as in Figure S1F)

- $p_D = \frac{h^N}{(d_{FP}^N + h^N)}(\frac{\pi}{a_V})(\frac{t_V}{t_{MIN}})$
  where N determines how steeply predator detection decreases with distance to the predator d_{FP} (maximal distance is 50 meters), h is the distance at which predator detection probability is half maximal, and $\alpha = \frac{\pi}{a_V}\frac{t_V}{t_{MIN}}$ describes how predator detection varies with SCAN duration t_V and angle a_V (where $0 < a_V \leq 2\pi$ in radians). t_V evolves to become equal to t_{MIN}, and a_V evovles to be 2π so that $\alpha = 0.5$.

- Duration:
  - if (predator detected): random duration between 0 and t_V.
  - else: t_V

FLEE: flee to safety after detecting a predator

- no change in spatial position, safety is simply assumed
- duration: REACTIONTIME

1.7.3 Forager discrete updates

Each minute for all foragers:

- Age: age + 1 minute
- Energy: $e_i = e_i - E_m$
  where E_m is energy metabolism per minute
- Waiting time (after fleeing from predator):
  - if (in state SAFE): waiting time: waiting time - 1 minute
  - if (waiting time == 0) state = NORMAL
- Reproduction:
  - if (population > 10 and $e_i >= E_M$): enough energy to give birth
    where e_i is forager's energy level, and E_M is energy needed to give birth.
    * Energy change due to birth: $e_i = e_i - E_M/2$
    * Offspring energy: $E_M/2$
    * Inheritance: offspring inherits all evolvable parameters (see Table 1, main text).
    * Mutation: with probability 0.05 each "gene" (evolvable parameter) is mutated. A new value is drawn from a normal distribution about the parent's parameter value within an preset range (see Table 1).
  - if (population < 10): forced birth
    * probability to choose parent: $(e_i/\sum e_i)^n$ where $n(=5)$ scales selection of the parent with most energy.
    * no energy costs for birth
- Death (forager dies if):
  - $e_i \leq 0$
  - age >= maximum age
  - RAND < death rate (equivalent to 0.1 per year)
  - caught by predator

1.7.4 Predator decision making

As for foragers, predators are in event scheduling list. If soonest ending action is of a predator then the following scheme is used:

- Complete current action:
  - ATTACK: get new position.
    If predator reaches forager before the forager completes FLEE then the predator catches forager and forager dies.
  - MOVE: get new position
- Choose new action:
  - if (any foragers < 50 meters): choose closest as target
    - if (target < d_P meters): ATTACK
    - if (target FLEEs): ATTACK (actually redundant because it is too late to catch the target)
    - else (if target too far and not FLEEing): MOVE in direction of target
  - else (no targets): MOVE forward in current heading

1.7.5 Predator actions

The predator actions update the predators position:
MOVE:

- Get new position and make sure it is on the grid:
  - $\vec{p'} = d_M\vec{v} + \vec{p}$
    where d_M is the MOVE distance = 1.
  - while $\bar{(}p'_x \; or \; p'_y < 0$ or $p'_x \; or \; p'_y >= \sqrt{A})$
    - $\vec{v}$ = normalized random vector
    - $\vec{p'} = d_M\vec{v} + \vec{p}$
- Duration: $d_M * t_M$
  where t_M is the time it takes the forager to move 1 meter.

ATTACK: essentially the same as MOVE but

- $\vec{p'} = \vec{f}$ where $\vec{f}$ is the position of target forager and $\vec{p}$ the position of the predator
- duration: distance to forager * predator attack speed
  (Note that if ATTACK was stimulated by forager fleeing, duration has in addition "reactiontime" added to it).

1.7.6 Resources

For all depleted resource items every minute:

- all depleted resource items with this minute of the year as appearance / regrow time, re-appear.
- resource items that are eaten disappear immediately.

References

1. Grimm V, Berger U, Bastiansen F, Eliassen S, Ginot V, et al. (2006) A standard protocol for describing individual-based and agent-based models. Ecological modelling 198: 115–126.

2. van der Post DJ, Semmann D (2011) Local orientation and the evolution of foraging: Changes in decision making can eliminate evolutionary trade-offs. PLoS Comput Biol 7: e1002186.

3. van der Post DJ, Semmann D (2011) Patch depletion, niche structuring and the evolution of cooperative foraging. BMC Evolutionary Biology 11: 335.

4. Hogeweg P, Hesper B (1985) Socioinformatic processes: MIRROR modelling methodology. J Theor Biol 113: 311–330.

5. Hogeweg P, Hesper B (1991) Evolution as pattern processing: TODO as substrate for evolution. In: Meyer JA, Wilson SW, editors, Proceedings of the first international conference on simulation of adaptive behavior on From animals to animats, Cambridge, Mass.: MIT Press. pp. 492–497.
