# Supplementary material for: The Evolution of Different Forms of Sociality: Behavioral Mechanisms and Eco-Evolutionary Feedback
Source: PLoS One. 2015 Jan 28;10(1):e0117027. doi: 10.1371/journal.pone.0117027 (PMC4309640; doi:10.1371/journal.pone.0117027)
Supplement: S2 Text — (PDF) [file pone.0117027.s002.pdf]

Text S2: Evolved vigilance and foraging parameters

Daniel J. van der Post[1,2,3,*], Rineke Verbrugge[1], Charlotte K. Hemelrijk[2]
1 Institute of Artificial Intelligence, University of Groningen, P. O. Box 407, 9700 AK, Groningen, The Netherlands
2 Behavioural Ecology and Self-Organization, University of Groningen, P. O. Box 11103, 9700 CC Groningen, The Netherlands
3 Centre for Social Learning and Cognitive Evolution, School of Biology, University of St. Andrews, Queens Terrace, St. Andrews, Fife KY16 9TS, United Kingdom
∗ **E-mail: d.j.vanderpost@gmail.com**

In Figure 1 we show the evolved vigilance and foraging parameters in populations of both solitary (blue) and grouping (orange) foraging for different predation risk (d_P). The main result is that when vigilance p_V evolves (A: $d_P = 5 - 7$; Figure 2A, main text), the vigilance angle a_V (D) evolves to its maximal value, to ensure the predator is seen when it approaches from behind. Vigilance duration t_V evolves to its minimal value (G), so that scanning for the predator occurs "briefly and often", rather than "rarely and long" (stars show where vigilance did not evolve). Moreover, the time that foragers wait before resuming foraging after fleeing from a predator t_P (see Figure S1E, in Text S1), evolves to be greater than about 50 minutes (I), which ensures that the predator is out of detection range and does not immediately return when foraging is resumed. With respect to foraging parameters, we find that when vigilance evolves, there is a switch from having low move probabilities and long move distances, to high move probabilities and short move distances (compare no predator and $d_P = 9$ with $d_P = 5 - 7$ in B and C). Long move distances evolve so that searching for food in the same place is avoided [1], however this can also be achieved by repeating movement with a greater move probability p_M. In combination with vigilance, the latter option has preference because this enables more regular scanning for the predator during movement. When vigilance disappears in large groups ($d_P = 0$), again long move distances evolve. Otherwise, as we found previously without vigilance [1], the move angle a_M does not evolve (E), and the food scan angle a_F evolves to around 250 degrees, which is where food item detection is maximal. Moreover, movement duration t_M (H) and foraging duration t_F (not shown, but results identical to those of t_M), evolve to minimal values. Qualitatively, foraging is therefore the same as without vigilance or grouping.

References

1. van der Post DJ, Semmann D (2011) Local orientation and the evolution of foraging: Changes in decision making can eliminate evolutionary trade-offs. PLoS Comput Biol 7: e1002186.

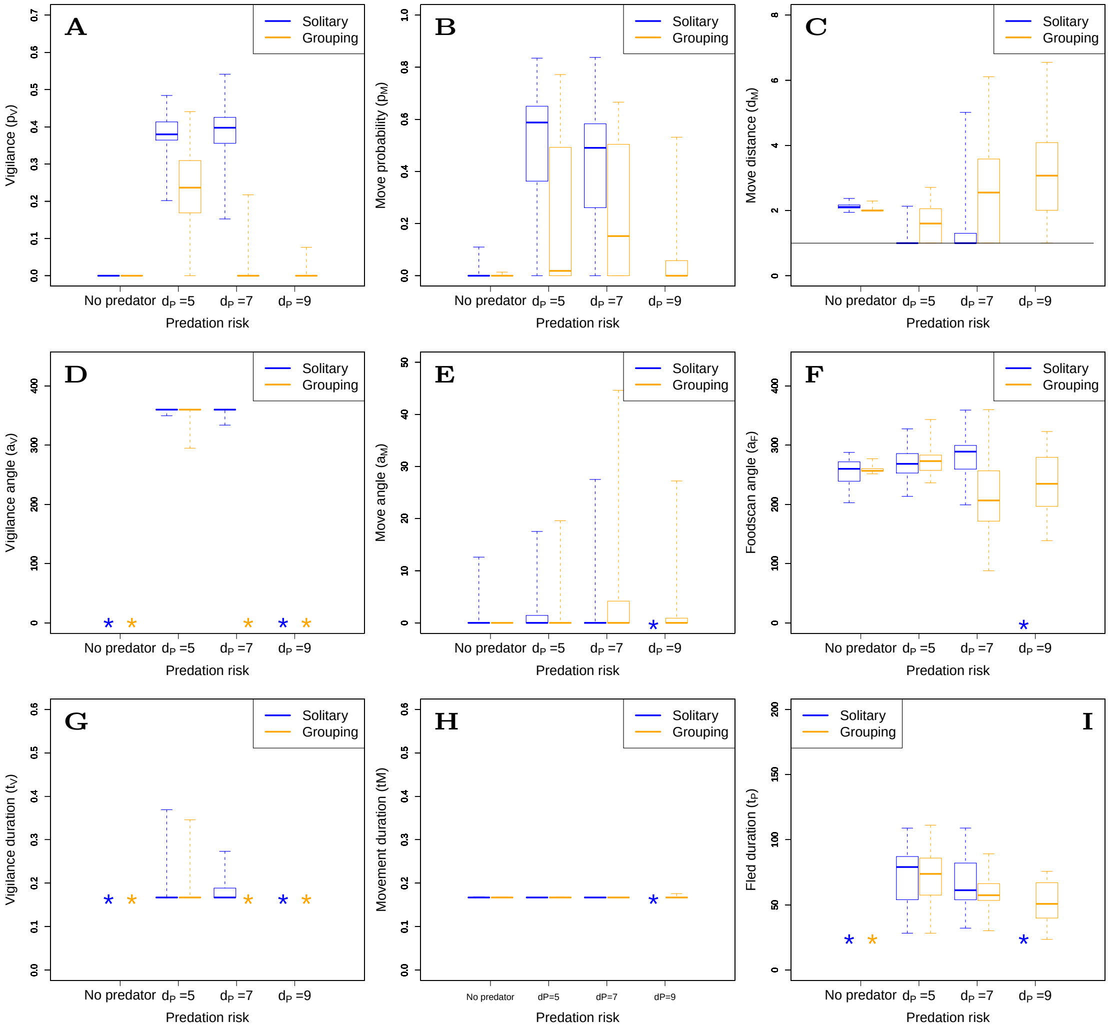

Figure 1. Evolved vigilance and foraging parameters for different predation pressure (d_P) in solitary (blue) and grouping (orange) populations. A: vigilance rate p_V; B: move probability p_M; C: move distance d_M; D: vigilance angle a_V; E: move angle a_M; F: foodscan angle a_F; G: vigilance duration t_V; H: movement duration t_M; I: fled duration t_P. Shown are box plots of ancestors between year 800 and 900, with median, upper and lower quartiles and max-min range. Blue stars at $d_P = 9$ indicate either (i) no predation, or (ii) that the solitary population is not viable at the highest predation risk ($d_P = 9$).
