# Supplementary material for: The Evolution of Different Forms of Sociality: Behavioral Mechanisms and Eco-Evolutionary Feedback
Source: PLoS One. 2015 Jan 28;10(1):e0117027. doi: 10.1371/journal.pone.0117027 (PMC4309640; doi:10.1371/journal.pone.0117027)
Supplement: S3 Text — (PDF) [file pone.0117027.s003.pdf]

Text S3: Evolved grouping parameters

Daniel J. van der Post[1,2,3,*], Rineke Verbrugge[1], Charlotte K. Hemelrijk[2]
1 Institute of Artificial Intelligence, University of Groningen, P. O. Box 407, 9700 AK, Groningen, The Netherlands
2 Behavioural Ecology and Self-Organization, University of Groningen, P. O. Box 11103, 9700 CC Groningen, The Netherlands
3 Centre for Social Learning and Cognitive Evolution, School of Biology, University of St. Andrews, Queens Terrace, St. Andrews, Fife KY16 9TS, United Kingdom
∗ E-mail: d.j.vanderpost@gmail.com

In Figure 1 we show the evolved grouping parameters for different predation risk d_P. Larger groups evolve as predation risk increases (D and Figure 2A main text, blue). Larger group sizes result from a combination of a greater number of neighbors being tolerated n_R (A), and smaller repulsion zones (E). There is also a tendency for foragers that form large groups to have large attraction angles (C) and attraction zones (F). Overall, foragers do not evolve repulsion angles (B), unless the repulsion zone evolves to zero (compare B to E), in which case the repulsion angle has no function. Note that when there is no repulsion zone, foragers still move apart during foraging, since when they move to food they do not take the position of their neighbors into account.

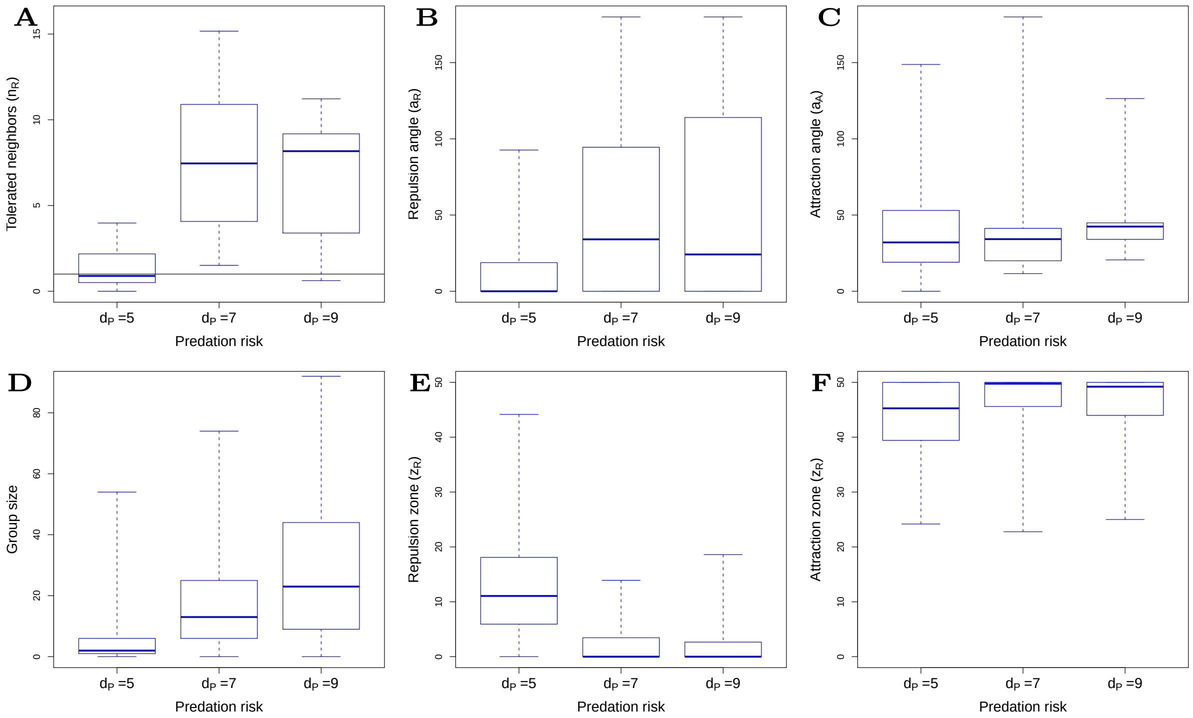

Figure 1. Evolved grouping parameters for different predation risk (d_P). A: tolerate neighbors n_R; B: repulsion turning angle a_R; C: attraction turning angle a_A; D: group size; E: repulsion zone z_R; F: attraction zone z_A. Box plots are shown for ancestor traces between year 800 and 900, with median, upper and lower quartiles and max-min range.
