# Supplementary material for: The Evolution of Different Forms of Sociality: Behavioral Mechanisms and Eco-Evolutionary Feedback
Source: PLoS One. 2015 Jan 28;10(1):e0117027. doi: 10.1371/journal.pone.0117027 (PMC4309640; doi:10.1371/journal.pone.0117027)
Supplement: S4 Text — (PDF) [file pone.0117027.s004.pdf]

Text S4: Dilution of risk and contagious fleeing

Daniel J. van der Post[1,2,3,*], Rineke Verbrugge[1], Charlotte K. Hemelrijk[2]
1 Institute of Artificial Intelligence, University of Groningen, P. O. Box 407, 9700 AK, Groningen, The Netherlands
2 Behavioural Ecology and Self-Organization, University of Groningen, P. O. Box 11103, 9700 CC Groningen, The Netherlands
3 Centre for Social Learning and Cognitive Evolution, School of Biology, University of St. Andrews, Queens Terrace, St. Andrews, Fife KY16 9TS, United Kingdom
∗ E-mail: d.j.vanderpost@gmail.com

In Figure 1 we show how grouping can reduce predation rates via the dilution of risk (A) and via information transmission through contagious fleeing (B). Grouping can reduce predation rates via the encounter-dilution effect [1, 2], where every additional group member dilutes predation risk relative to that experienced by solitary foragers: r_S/N, where r_S is the risk of solitary foragers and N is group size (dashed line, where the risk of solitary foragers (left-most red dot) was measured in populations without vigilance). However, this is an idealized scenario, which assumes that the rate of encountering predators is the same for solitary foragers and groups. We therefore measured predation rates (red dots) in populations of foragers that form groups of different sizes (as found in our evolutionary simulations), but without any vigilance. We find that predation rates on groups (red dots) are somewhat greater than idealized dilution (dashed line), and conclude that groups do encounter predators more often than solitary foragers do. However, this does not make the effect of dilution obsolete, otherwise death rates in groups would be equal to the per capita predation rate on solitary foragers (red dots on solid grey line). Thus, the encounter-dilution effect reduces predation rates in our model.

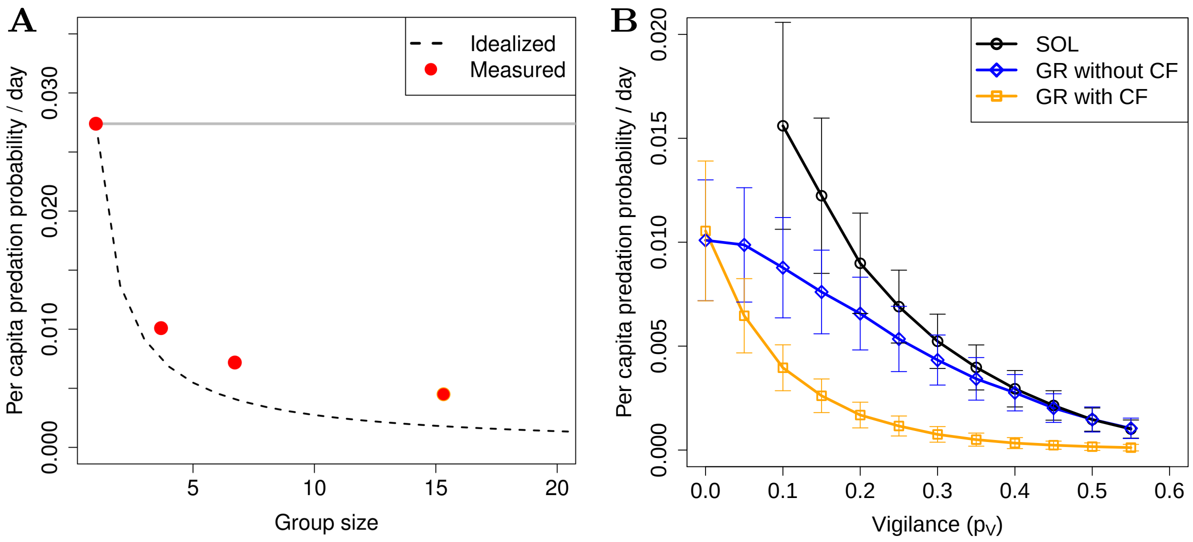

Figure 1. Dilution of risk (A) and contagious fleeing (B). A: Average per capita predation rates in groups of different sizes without vigilance (red dots), compared to the idealized dilution of risk extrapolated from the rate of predation in solitary foragers r_S as r_S/N (dashed line). B: Average rates of predation as a function of vigilance rate in solitary (black), groups without contagious fleeing (without CF, blue), and groups with contagious fleeing (with CF, orange). Simulations with grouping had an average group size of about 5 individuals. Whiskers are standard deviation. Solitary populations are inviable for the lowest two vigilance rates ($p_V \leq 0.05$).

To determine the effect of contagious fleeing, where one individual detects the predator and flees and stimulates its neighbors to flee, we measured deaths due to predation as a function of vigilance rates (B). We used simulations with fixed populations with either solitary foragers, or foragers with grouping parameters that generate groups of about 5 individuals and $d_P = 5$. For populations that form groups, we compared populations with contagious fleeing (orange), and without (blue), in order to tease apart the contributions of the encounter-dilution effect (A) and contagious fleeing. Relative to solitary foragers, predation risk is reduced in groups without contagious fleeing, although mainly at low vigilance values (compare black to blue line). In groups with contagious fleeing (orange), predation rates are reduced even further. This happens because information about predators obtained with vigilance is spread via contagious fleeing. As a result, the response of the group as a whole becomes equal to the response of the first individual to detect the predator. Thus, in addition to encounter-dilution, contagious fleeing reduces predation risk in our model.

References

1. Turner G, Pitcher T (1986) Attack abatement: A model for group protection by combined avoidance and dilution. American Naturalist 128: 228–240.

2. Mooring M, Hart B (1992) Animal grouping for protection from parasites: Selfish herd and encounter-dilution effects. Behaviour 123: 173–193.
