# Supplementary material for: The Evolution of Different Forms of Sociality: Behavioral Mechanisms and Eco-Evolutionary Feedback
Source: PLoS One. 2015 Jan 28;10(1):e0117027. doi: 10.1371/journal.pone.0117027 (PMC4309640; doi:10.1371/journal.pone.0117027)
Supplement: S5 Text — (PDF) [file pone.0117027.s005.pdf]

Text S5: Constraints on group size evolution

Daniel J. van der Post[1,2,3,*], Rineke Verbrugge[1], Charlotte K. Hemelrijk[2]
1 Institute of Artificial Intelligence, University of Groningen, P. O. Box 407, 9700 AK, Groningen, The Netherlands
2 Behavioural Ecology and Self-Organization, University of Groningen, P. O. Box 11103, 9700 CC Groningen, The Netherlands
3 Centre for Social Learning and Cognitive Evolution, School of Biology, University of St. Andrews, Queens Terrace, St. Andrews, Fife KY16 9TS, United Kingdom
∗ E-mail: d.j.vanderpost@gmail.com

If coordinated travel depends on the leader-follower system, then one would expect evolutionary constraints on groups size, because large groups either get stuck in space (Video S5), or split up into a number of pairs (Video S6). Such constraints would explain the period of evolutionary stasis in group size (Figure 2B, phase II, main text). To test this explicitly, we studied the evolution of group size in populations from year 140 (middle of phase II, representing a population dependent on leader-follower travel), and year 330 (at the start of phase III, representing a population recently independent of leader-follower travel).

We ran simulations where vigilance was preset and fixed at a certain value in range from 0.15 to 0.5, representing a reasonable range around the vigilance values that predominate in phase II (roughly 0.3, Figure 2B, orange, main text), and where values lower than 0.3 represent an evolutionary stimulus to evolve larger groups. We then study how populations from year 140 and 330 respond to this stimulus. The simulations were run for 100 years, where vigilance p_V was fixed but all other evolvable parameters

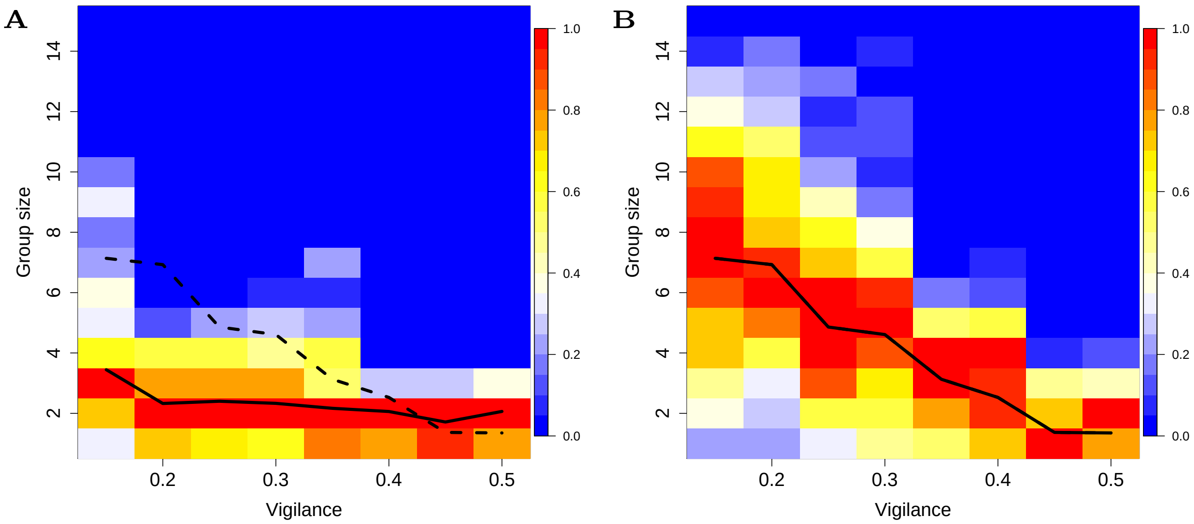

Figure 1. Constraints on group size evolution. Heat maps showing fitness of foragers in different groups sizes as a function of preset and fixed vigilance rates p_V for populations from year 140 (A), and year 320 (B), from the simulation in Figure 2B, main text. The simulations were run for 100 years, and grouping parameters could evolve freely. Only data from the last 55 years, because during this time the evolved group size was relatively stable. From each simulation we measured average evolved group size to obtain the solid lines in A and B (to ease comparison we plot the solid line of B as a dashed line in A). For the fitness landscape we binned individuals in terms of their lifetime average group size (at intervals of 1) and calculated average number of offspring per bin (fitness). We then normalized over bins (i.e. all bins divided by maximum fitness) such that different simulations (p_V) could be directly compared (i.e. each column in heat map of A and B was normalized).

could evolve. We then derive fitness landscapes for populations from the two time points. We used data from the last 55 years, because visual inspection revealed that during this time the evolved group size was relatively stable. From each simulation we measured average evolved group size to obtain the solid lines in A and B (to ease comparison we plot the solid line of B as a dashed line in A). For the fitness landscape we binned individuals in terms of their lifetime average group size (at intervals of 1) and calculated average number of offspring per bin (fitness). We then normalized over bins (i.e. all bins divided by maximum fitness) such that different simulations (p_V) could be directly compared (i.e. each column in heat map of A and B was normalized).

Results are shown in Figure 1. For populations from year 140 (A), fitness drops off rapidly as group size increases, and group sizes are constrained to small values. For populations at year 330, maximal fitness is found at larger group sizes (A, compared dashed to dotted line), and there is a wider range of group sizes with high fitness (B). This reveals that the difference in evolved group sizes (black lines) is due to structural differences in the fitness landscape of the areas in parameter space where the two populations are located, and not just differences in initial group size. Thus, in populations that are dependent on leader-follower travel, group sizes are constrained to relatively small values.
