# Supplementary material for: The Evolution of Different Forms of Sociality: Behavioral Mechanisms and Eco-Evolutionary Feedback
Source: PLoS One. 2015 Jan 28;10(1):e0117027. doi: 10.1371/journal.pone.0117027 (PMC4309640; doi:10.1371/journal.pone.0117027)
Supplement: S6 Text — (PDF) [file pone.0117027.s006.pdf]

Text S6: Evolution of repulsion and attraction in leaders and followers

Daniel J. van der Post[1,2,3,*], Rineke Verbrugge[1], Charlotte K. Hemelrijk[2]
1 Institute of Artificial Intelligence, University of Groningen, P. O. Box 407, 9700 AK, Groningen, The Netherlands
2 Behavioural Ecology and Self-Organization, University of Groningen, P. O. Box 11103, 9700 CC Groningen, The Netherlands
3 Centre for Social Learning and Cognitive Evolution, School of Biology, University of St. Andrews, Queens Terrace, St. Andrews, Fife KY16 9TS, United Kingdom
∗ E-mail: d.j.vanderpost@gmail.com

In Figure 1 we show the evolutionary changes in repulsion and attraction turning angle during phase II (leader-follower societies). For the repulsion turning angle, the population starts off (at the origin of grouping: year 70) clustered mainly on a value of about 0.4. Thereafter the population quickly splits into two lineages, a dominant lineage without a repulsion turning angle of zero (orange line: follower lineage, which is the overall line of descent), and a second lineage with greater values (lineage b, first grey line: leader lineage). Later, new leader lineages (c and d) branch off from the follower lineage, each time replacing existing leader lineages. A repulsion turning angle of zero is a prerequisite for independence from leader-follower travel. The attraction turning angle does not show clear evolutionary branching. Instead, there is a general decline in the attraction turning angle over time. Once the attraction turning angle is low enough, followers can become independent of leaders.

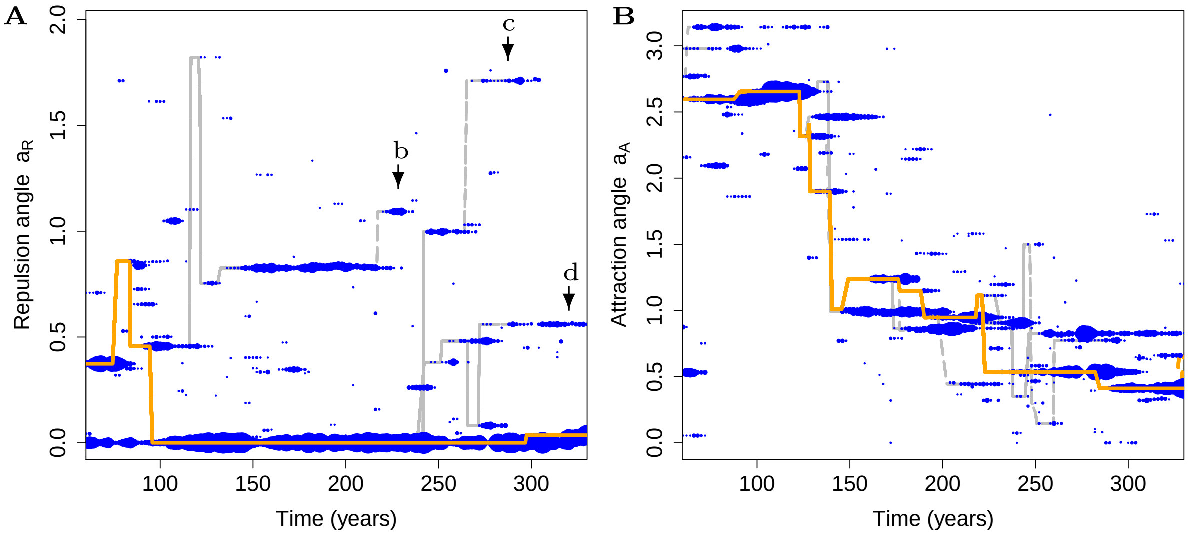

Figure 1. Leaders and followers: evolution of repulsion (A) and attraction (B) turning angles. Blue circles show the distribution on the parameter in the population, where circle size represents the number of individuals (at least 5) with that parameter value. Circles can overlap. The orange line shows the overall line of descent. Grey lines show the progression in different lineages present every 20 years, where the last 20 ancestors are not shown in order to prune out short lived lineages. Data taken from the simulation shown in Figure 2B, main text. The lineages b, c and d shown in Figure 3A (main text) are indicated.
