# Supplementary material for: The Evolution of Different Forms of Sociality: Behavioral Mechanisms and Eco-Evolutionary Feedback
Source: PLoS One. 2015 Jan 28;10(1):e0117027. doi: 10.1371/journal.pone.0117027 (PMC4309640; doi:10.1371/journal.pone.0117027)
Supplement: S7 Text — (PDF) [file pone.0117027.s007.pdf]

Text S7: Co-existence of vigilant and non-vigilant foragers and the survival advantage of vigilant foragers

Daniel J. van der Post[1,2,3,*], Rineke Verbrugge[1], Charlotte K. Hemelrijk[2]
1 Institute of Artificial Intelligence, University of Groningen, P. O. Box 407, 9700 AK, Groningen, The Netherlands
2 Behavioural Ecology and Self-Organization, University of Groningen, P. O. Box 11103, 9700 CC Groningen, The Netherlands
3 Centre for Social Learning and Cognitive Evolution, School of Biology, University of St. Andrews, Queens Terrace, St. Andrews, Fife KY16 9TS, United Kingdom
$\ast$ **E-mail: d.j.vanderpost@gmail.com**

To characterize the social-ecological conditions for the social niche of non-vigilant foragers, we ran two kinds of simulations without mutations to obtain the fitness landscape shown in Figure 1A: (i) To determine how population size depended on the proportion of vigilant foragers (solid line), we ran simulations with a fixed proportion of vigilant foragers ranging from 0.0 to 0.95 at intervals of 0.125: we let a vigilant forager be born when the proportion was too low, and vice-versa. For this we used the vigilant and non-vigilant genotypes that we found to co-exist (Figure 4A, main text). Simulations were run for 17 years (2 year start-up period). We measured the average population size over the last 15 years. (ii) To determine when vigilant or non-vigilant foragers had a fitness advantage (heatmap), we ran simulations with fixed proportions and fixed populations. Simulations were as in (i), except that a forager of the same type (vigilant or not) was born whenever a forager of a certain type died. We varied populations from 40 to 120 with intervals of 10, and the following selection function was used: $p_i = e_i^n/(\sum_{j=0}^{N} e_j)^n$, where p is the probability that a forager i reproduces, e_i is a forager's energy, N is the population size, and $n(= 3)$ is the selection co-efficient. Note that since parents loose half their energy after reproducing, birth rates to depend on food intake. We measured the average lifetime reproductive success (LRS) of vigilance and non-vigilant foragers (last 15 years of simulations), and plot the difference between them.

The resulting fitness landscape reveals where non-vigilant foragers have a fitness advantage (blue zone), i.e. the conditions for a social niche for non-vigilance: large populations with a high proportion of vigilant foragers (top right). Under such conditions, non-vigilant foragers can invade, and the proportion of vigilant foragers will decline (downward arrow). As a result, vigilance is reduced and the population size will decrease (left arrow). The population decrease causes group size to decline (see Figure 4B, main text). In combination, decreased group size and a decreased proportion of vigilant foragers reduces the advantage of non-vigilant foragers (see below). This continues until the fitness advantage of non-vigilant foragers disappears (dashed line), and population size and the proportion of vigilant foragers equilibrates: a frequency-, density-, and group-size-dependent co-existence.

In Figure 1B we show how the survival advantage of vigilant foragers relative to non-vigilant ones, declines as group size and proportion of vigilant foragers in the population increases (main graph). These results are based on a highly reduced model: If one assumes that groups form randomly, then the average probability of having vigilant neighbors can be represented as $P_v[G-1]$, where G is group size, and P_v is the proportion of vigilant foragers in the population. The vigilance experienced is therefore $V_{G_n} = P_v[G-1]$, for non-vigilant foragers (orange, inset), and $V_{G_v} = P_v[G-1]+1$, for vigilant foragers (blue, inset).

By not contributing, non-vigilant foragers reduce vigilance in groups, while vigilant foragers live in groups with more vigilance (compare blue to orange, inset). Vigilant foragers therefore have a survival advantage, $(P_v[G-1]+1)/P_v[G-1]$ (main graph; blue divided by orange, inset), but this depends on group size (from left to right, main graph). The advantage is maximal when foragers are solitary ($G = 1$), but disappears when groups become very large. Moreover, the advantage is greater when the proportion of vigilant individuals P_v is low (going from bottom to top line, main graph), because then the probability that non-vigilant foragers find themselves in a group with vigilance is lower. Note that in our model groups do not form randomly, and assortment increases the survival advantage of vigilant foragers.

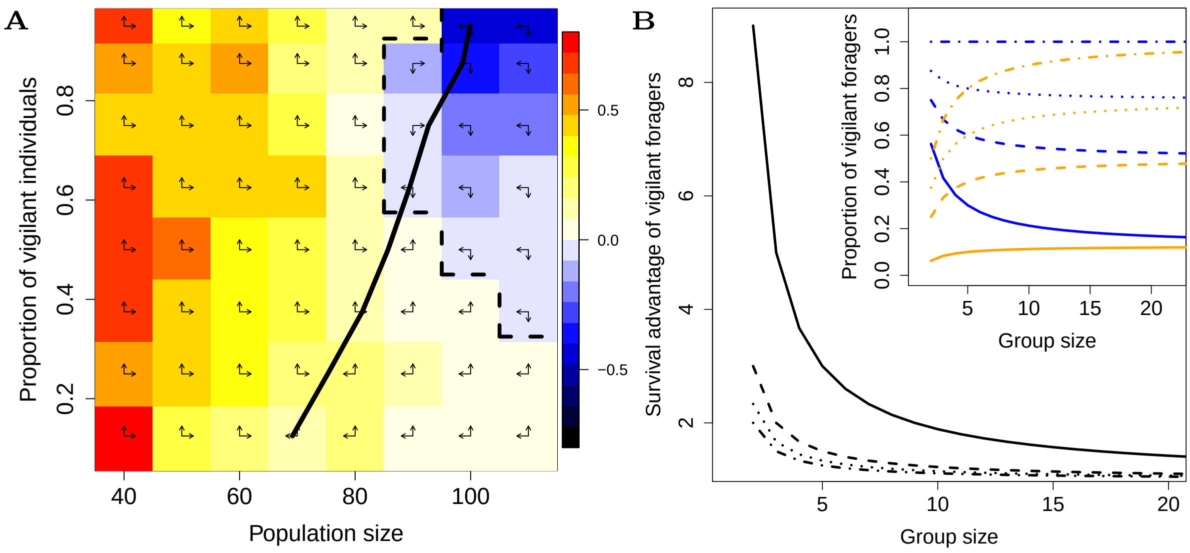

Figure 1. Co-existence and the survival advantage of vigilant foragers. A: Fitness landscape showing where non-vigilant (blue) and vigilant (red) have greater fitness according to changes in population size and the proportion of vigilant foragers in the population. Vertical arrows indicate how fitness will affect the proportion of vigilant foragers. The solid line indicates the average, or equilibrium, population size for a given proportion of vigilant foragers. Horizontal arrows indicate how the population size would change from a particular combination of population size and proportion of vigilant foragers. Please see the text for an explanation of the types of simulations used to generate this figure. B: The survival advantage of vigilant foragers as a function of group size based on a simple model (see text) for different proportions of vigilant foragers in the population. Inset: The proportion of vigilant foragers (including the forager itself) that vigilant (blue) and non-vigilant (orange) foragers experience in the group they partake in, as a function of group size and for different proportions of vigilant foragers in the population. Proportions of vigilant foragers: 0.15 (solid); 0.5 (dashed); 0.75 (dotted); 1.0 (dot-dashed).
