# Supplementary material for: The Evolution of Different Forms of Sociality: Behavioral Mechanisms and Eco-Evolutionary Feedback
Source: PLoS One. 2015 Jan 28;10(1):e0117027. doi: 10.1371/journal.pone.0117027 (PMC4309640; doi:10.1371/journal.pone.0117027)
Supplement: S8 Text — (PDF) [file pone.0117027.s008.pdf]

Text S8: Controlled evolutionary simulation

Daniel J. van der Post[1,2,3,*], Rineke Verbrugge[1], Charlotte K. Hemelrijk[2]
1 Institute of Artificial Intelligence, University of Groningen, P. O. Box 407, 9700 AK, Groningen, The Netherlands
2 Behavioural Ecology and Self-Organization, University of Groningen, P. O. Box 11103, 9700 CC Groningen, The Netherlands
3 Centre for Social Learning and Cognitive Evolution, School of Biology, University of St. Andrews, Queens Terrace, St. Andrews, Fife KY16 9TS, United Kingdom
*** E-mail: d.j.vanderpost@gmail.com**

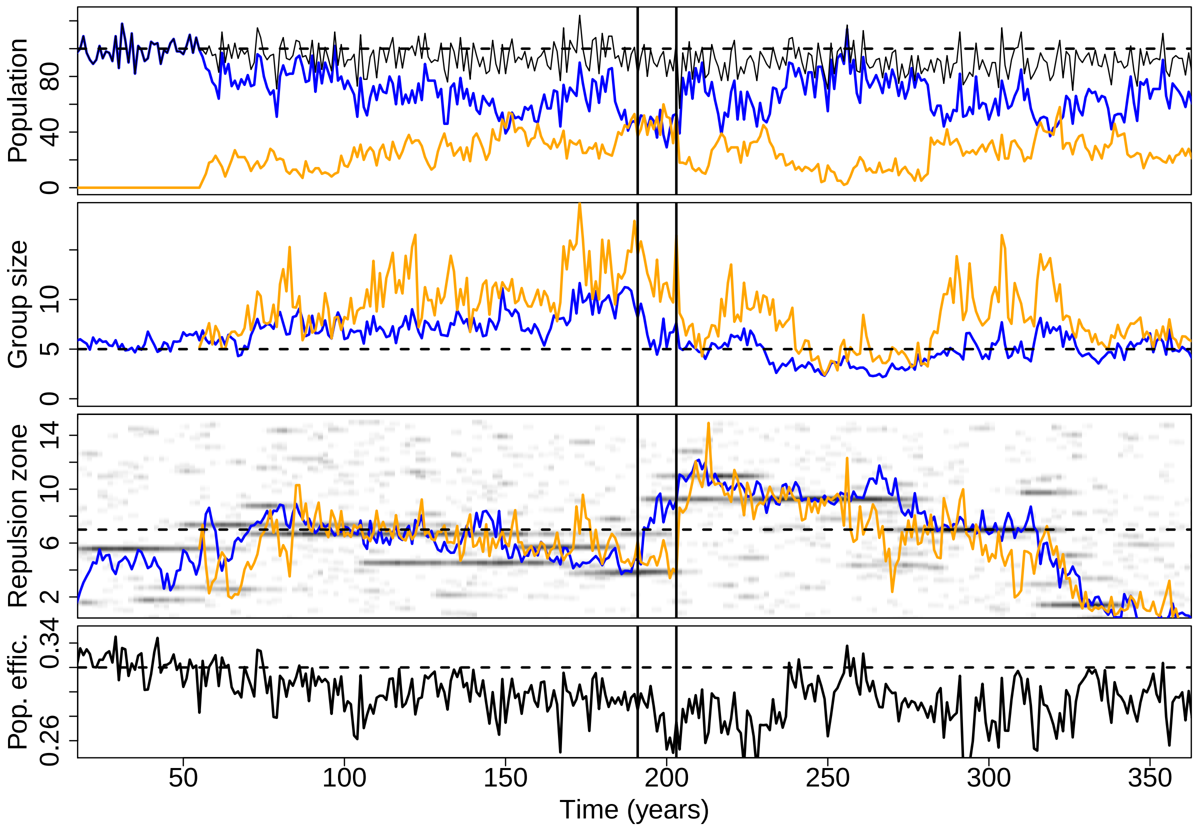

Figure 1. Timeplot of controlled evolutionary simulation where non-vigilant foragers (orange) invade a vigilant forager (blue) population. Here vigilance is initially fixed at $p_V = 0.15$, and only from year 55 are mutations allowed between $p_V = 0.15$ and $p_V = 0.0$. Grouping parameters can evolve freely throughout. Top: population size; Middle-top: average group size; Middle-bottom: average (solid lines) and population distribution (grey coloring) of repulsion zone z_R; Bottom: population efficiency (reflecting depletion of resources in the environment).

In Figure 1 we show population size (top), group size (middle-top), repulsion zone (middle-bottom) and population efficiency (bottom) of a controlled evolutionary simulation. Population efficiency is calculated as the deviation of average resource density from the maximal resource density (0.535), where a greater level of resource depletion corresponds to a greater population efficiency.

Vigilance is initially (first 50 years after 5 year start-up period) fixed at $p_V = 0.15$, but all other

parameters can evolve freely. During this period group size evolves to a stable level, i.e. optimized group sizes. After year 50, vigilance can mutate between $p_V = 0.15$ and $p_V = 0.0$, and non-vigilant lineages invade the population (orange, top) and average group size increases (orange and blue, middle-top). This increase in group size corresponds to a decrease in values of the repulsion zone (orange and blue, middle-bottom). At the same time the population efficiency (how well the population depletes the environment) declines (bottom). One reason is reduced lifespan and therewith smaller populations, the other is that local foraging competition increases as group become larger, reducing overall foraging efficiency.

In the period from year 190 to 203 (between vertical lines), new "small group" lineages arise with large repulsion zones, as indicated by the horizontal grey lines and the increase in average repulsion zone (blue, middle-bottom). This period corresponds with an increase in group size differentiation between vigilant and non-vigilant foragers. This differentiation can be illustrated by a negative correlation between group size and vigilance. This correlation is typically negative because non-vigilant foragers have stronger grouping tendencies than vigilant ones. Thus, at year 185 the correlation coefficient is $r = -0.2633816$ ($CI : -0.44148914, -0.06530514$). After differentiation in grouping tendencies, at year 195 the negative correlation is much stronger at $r = -0.5782023$ ($CI : -0.6961525, -0.4298559$), indicating a stronger degree of assortment of vigilant and non-vigilant foragers into different group sizes. By year 203, the "large group" lineages with small repulsion zones go extinct (middle-bottom, lowest grey lines), group sizes to decline (middle-top), and the population of vigilant foragers increases (top, blue). As a result, by year 205, the negative correlation between group size and vigilance has reduced to $r = -0.2962462$ ($CI : -0.4680376, -0.1028795$), indicating that differences in grouping tendency between vigilant and non-vigilant have diminished after year 195. By year 250, the vigilant population is again dominant (top, blue), and population efficiency is high (bottom). However, after year 250, group sizes start to increase again (middle top), and the repulsion zone starts to evolve to smaller values (middle-bottom), indicating a repeat of the process seen between years 100 and 190.
