# Supplementary material for: The Evolution of Different Forms of Sociality: Behavioral Mechanisms and Eco-Evolutionary Feedback
Source: PLoS One. 2015 Jan 28;10(1):e0117027. doi: 10.1371/journal.pone.0117027 (PMC4309640; doi:10.1371/journal.pone.0117027)
Supplement: S9 Text — (PDF) [file pone.0117027.s009.pdf]

Text S9: Assortment and grouping tendencies

Daniel J. van der Post[1,2,3,*], Rineke Verbrugge[1], Charlotte K. Hemelrijk[2]
1 Institute of Artificial Intelligence, University of Groningen, P. O. Box 407, 9700 AK, Groningen, The Netherlands
2 Behavioural Ecology and Self-Organization, University of Groningen, P. O. Box 11103, 9700 CC Groningen, The Netherlands
3 Centre for Social Learning and Cognitive Evolution, School of Biology, University of St. Andrews, Queens Terrace, St. Andrews, Fife KY16 9TS, United Kingdom
∗ E-mail: d.j.vanderpost@gmail.com

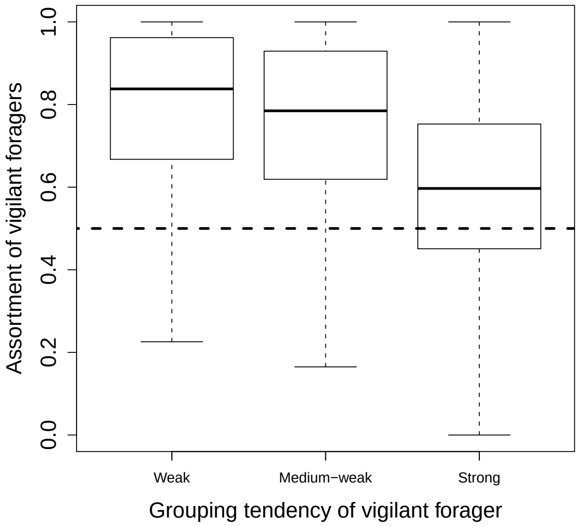

Figure 1. Assortment according to differences in grouping tendency. We measured the average lifetime assortment of vigilant foragers with other vigilant foragers (from year 5 - 50), where assortment a was measured every minute as $n_V/n_V + n_{NV}$, where n_V is the number of vigilant neighbors, and n_{NV} is the number of non-vigilant neighbors. In all simulations, non-vigilant foragers had strong grouping tendencies (group size of 10-30) and studied the assortment of vigilant foragers with three different grouping tendencies: weak (group size 2-5); medium-weak (group size 6-7); strong (group size 10-30).

In Figure 1 we show how differences in grouping tendencies affect assortment of vigilant and non-vigilant foragers into different groups. From the controlled evolutionary simulation (Text S8), we obtained the genotypes that remain after 50 years when mutations were stopped at year 100, 189 and 203. Thus we obtain a medium-weak grouping genotype (year 100), a strong grouping genotype (year 189, small repulsion zone), and a weak grouping genotype (year 203, large repulsion zone). We then ran simulations with fixed populations and a fixed proportion of 0.5 vigilant and 0.5 non-vigilant foragers (Text S7 for details of simulations with fixed populations and proportions). We measured the average lifetime assortment of vigilant foragers with other vigilant foragers (from year 5 - 50), where assortment a was measured every minute as $n_V/n_V + n_{NV}$, where n_V is the number of vigilant neighbors, and n_{NV} is the number of non-vigilant neighbors. We used non-vigilant foragers with strong grouping tendencies from year 189, and then ran three kinds of simulation: (i) vigilant foragers had medium-weak grouping tendencies (group sizes of 6-7 individuals) from the genotypes obtained at year 100; (ii) vigilant foragers

had strong grouping tendencies (group sizes of 10-30 individuals) from genotypes obtained at year 189; and (iii) vigilant foragers had weak grouping tendencies (group sizes of 2-5 individuals) from the genotypes obtained at year 203.

Results (boxplots) reveal that assortment depends on how weak grouping tendencies of vigilant foragers are relative to non-vigilant foragers with strong grouping tendencies. The least assortment occurs when vigilant foragers have strong grouping tendencies (right). The greatest assortment is when vigilant foragers have the weakest grouping tendencies (left). For the more intermediate case (medium-weak grouping tendencies) the assortment is intermediate to the other two cases (middle).
