# Supplementary material for: The Evolution of Different Forms of Sociality: Behavioral Mechanisms and Eco-Evolutionary Feedback
Source: PLoS One. 2015 Jan 28;10(1):e0117027. doi: 10.1371/journal.pone.0117027 (PMC4309640; doi:10.1371/journal.pone.0117027)
Supplement: S10 Text — (PDF) [file pone.0117027.s010.pdf]

Text S10: Optimal group size and increased predation

Daniel J. van der Post[1,2,3,*], Rineke Verbrugge[1], Charlotte K. Hemelrijk[2]
1 Institute of Artificial Intelligence, University of Groningen, P. O. Box 407, 9700 AK, Groningen, The Netherlands
2 Behavioural Ecology and Self-Organization, University of Groningen, P. O. Box 11103, 9700 CC Groningen, The Netherlands
3 Centre for Social Learning and Cognitive Evolution, School of Biology, University of St. Andrews, Queens Terrace, St. Andrews, Fife KY16 9TS, United Kingdom
∗ E-mail: d.j.vanderpost@gmail.com

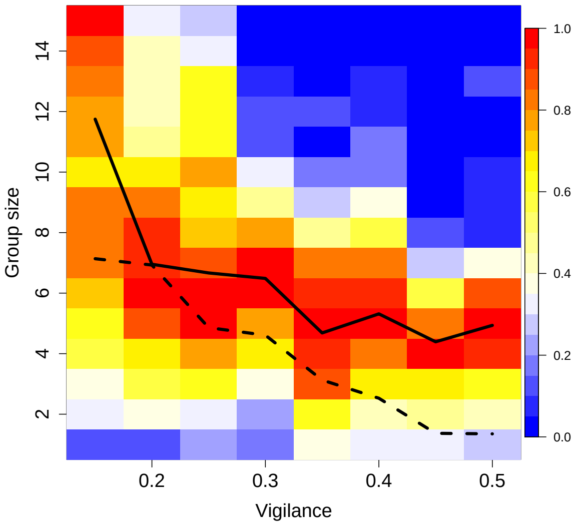

Figure 1. Optimal group size with increased predation risk. Heat maps showing fitness of foragers in different groups sizes as a function of preset and fixed vigilance rates p_V for a population from year year 320 from the simulation in Figure 2B (main text), with predation risk $d_P = 7$. The simulations were run for 100 years, and grouping parameters could evolve freely. Only data from the last 55 years, because during this time the evolved group size was relatively stable. From each simulation we measured average evolved group size to obtain the solid lines (to ease comparison we plot the solid line of Figure 1B (Text S5) as a dashed line). For the fitness landscape we binned individuals in terms of their lifetime average group size (at intervals of 1) and calculated average number of offspring per bin (fitness). We then normalized over bins (i.e. all bins divided by maximum fitness) such that different simulations (p_V) could be directly compared (i.e. each column in heat map of A and B was normalized).

In Figure 1 we show a group-size - vigilance fitness landscape for high predation risk (as in Figure 1B (Text S5), but then with predation risk $d_P = 7$), revealing that greater predation risk shifts optimal (red) and average group sizes (solid line) to larger values (compare heat map to that of Fig 1B (Text S5), $d_P = 5$; thick line of Figure 1B (Text S5) is shown here as dashed line). The heat map reveals that even when vigilance levels are high, small groups are not efficient (note low fitness (blue color) for small group sizes), and these cannot compete effectively with large groups, even if they are assorted.
