# Supplementary material for: The Evolution of Different Forms of Sociality: Behavioral Mechanisms and Eco-Evolutionary Feedback
Source: PLoS One. 2015 Jan 28;10(1):e0117027. doi: 10.1371/journal.pone.0117027 (PMC4309640; doi:10.1371/journal.pone.0117027)
Supplement: S11 Text — (PDF) [file pone.0117027.s011.pdf]

Text S11: Population size and mutation rate

Daniel J. van der Post[1,2,3,*], Rineke Verbrugge[1], Charlotte K. Hemelrijk[2]
1 Institute of Artificial Intelligence, University of Groningen, P. O. Box 407, 9700 AK, Groningen, The Netherlands
2 Behavioural Ecology and Self-Organization, University of Groningen, P. O. Box 11103, 9700 CC Groningen, The Netherlands
3 Centre for Social Learning and Cognitive Evolution, School of Biology, University of St. Andrews, Queens Terrace, St. Andrews, Fife KY16 9TS, United Kingdom
∗ E-mail: d.j.vanderpost@gmail.com

We varied the population size and mutation rate to investigate the impact on the co-evolutionary dynamics of group size and vigilance in phase III. The population size was doubled by increasing the area of the environment from 5.66 km^2 to 8 km^2, which doubled the carrying capacity of the environment and thus the population, but kept the population density constant. Since only a few simulations reached phase III within 1000 years in the simulations with a doubled population or low mutation rate, we initialized simulations with populations from phase III in the simulation shown in Fig. 2B (year 330), main text. All simulations that we discuss here concern $d_p = 5$.

In Fig. 1, we show average group size per time point in different evolutionary simulations. In Fig. 1A are the simulations of our standard setting with $d_p = 5$, with strong fluctuations of group size when simulations reach phase III. Here we focus on long-term fluctuations on the order of 50-100 years. Note that one simulation remains in phase I (solitary), and two in the phase II (leader-follower societies). When we increase the population size, fewer simulations reach phase III (Fig. 1B, III), but group size fluctuations appear large.

To standardize outcomes and systematically compare dynamics in phase III, we ran simulations that were initialized with populations from phase III (Fig. 1, C-F). In the standard settings (90-110 individuals, mutation rate 0.05), we again observe large group size fluctuations (Fig. 1, C), and two simulations revert to phase II and one simulation reverts to phase I. In a larger population (180-220 individuals, Fig. 1D), we observe similar dynamics, and there are no obvious differences. When we lower the mutation rate from 0.05 to 0.0125 (i.e. a four fold reduction), there is no obvious difference in group size fluctuations in the standard settings (Fig. 1, compare C to E), although with the lower mutation rate none of the simulations reverts to phase II or phase I (Fig. 1E). In the larger population however, the group size fluctuations appear to be reduced, with more stable group sizes in time, when the mutation rate is reduced (Fig. 1, compare D to F).

Thus it would appear that the combined effect of increasing population size and reducing mutation rate causes dynamics in phase III to stabilize. Both the increase in population size and the reduction of mutation rate can reduce stochasticity, indicating that stochasticity could play a role in the amplitude of fluctuations in group size. However, a reduced mutation rate could also impact the relative rates of ecological and evolutionary processes. On the one hand, as we reduce mutations it makes sense that eventually dynamics will be reduced simply because we limit opportunities for change in the model. On the other hand, the nature of the interaction between evolution and socio-ecological dynamics could be changing. A high mutation rate could affect the relationship between (i) the timescale at which new mutations are introduced into the population, and (ii) the timescale of grouping and population dynamics, which establish the selection pressures operating on individuals.

The issue of mutation rate raises the question of which mutation rate is reasonable. Our standard mutation rate (0.05) is high compared to most estimates. We chose this rate operationally, so that we observed groups evolving in a time period in the simulation that we could simulate within a reasonable period of real time. This operational choice for the mutation rate does not imply that it is unrealistic: it is not easy to determine what is a reasonable mutation rate for pseudo-genes. Pseudo-genes are so named because they represent higher-level phenotypic features of individuals, which are most likely coded for a by multitude of ‘real’ genes that interact in complex ways. Hence mutation of pseudo-genes represents a

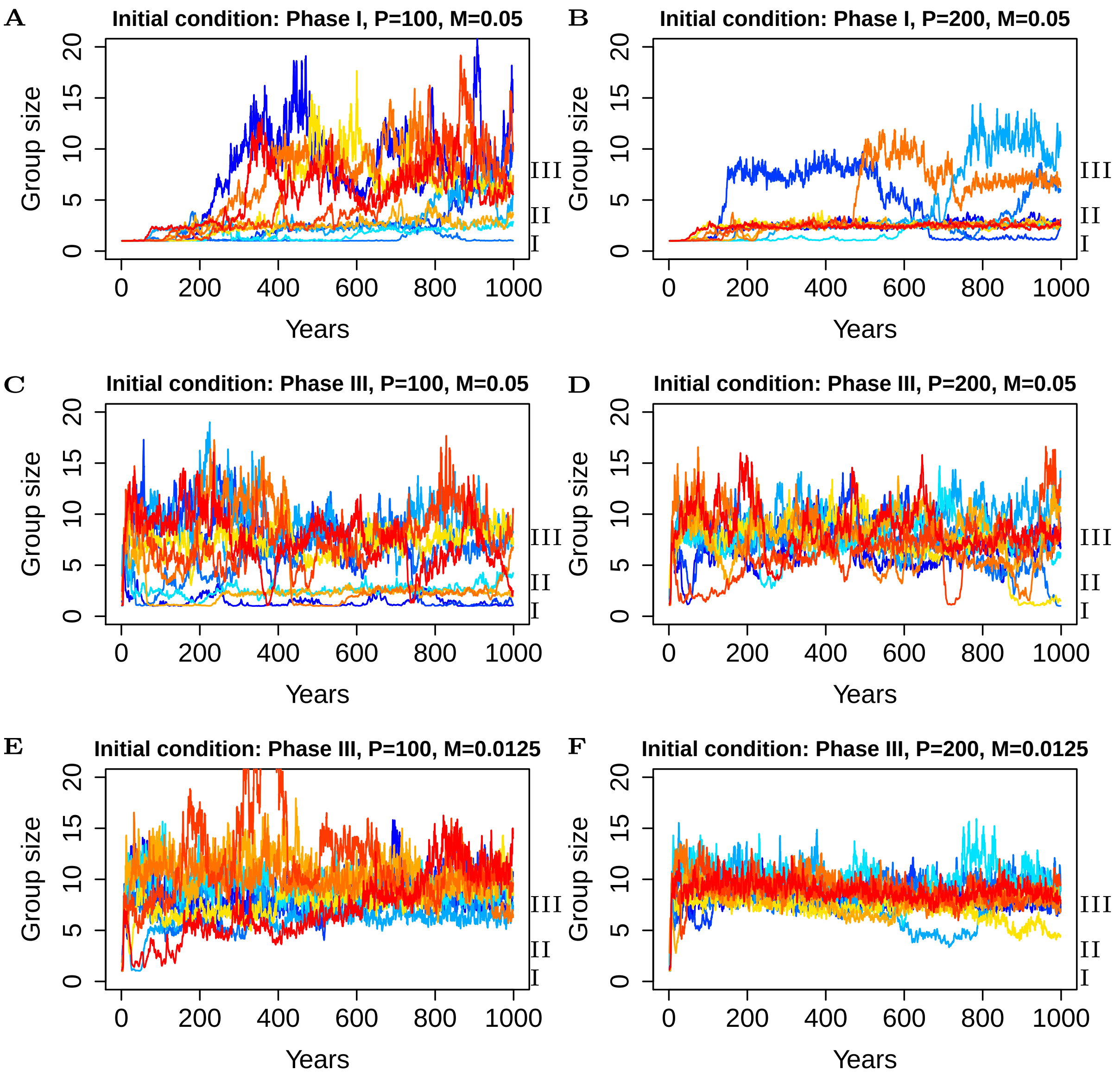

Figure 1. Effect of population size and mutation rate on phase III dynamics: timeplots. Average group size per time point in evolutionary simulations ($d_p = 5$). Smoothing: a running mean of 10 time points (every time point is a quarter of a year). A,C,E: standard population $P = 90 - 110$ individuals, 5.66 km^2); B,D,F: doubled population $P = 180 - 220$ individuals (8 km^2); A-B: starting with solitary individuals (phase I), mutation rate 0.05; C-D: starting with a population from phase III (year 330 in Fig. 2B main text), mutation rate 0.05; E-F: same as middle but with mutation rate 0.0125.

change in a higher-level phenotypic feature, where underlying genetic detail is implied, but not explicitly specified. Thus it seems reasonable to assume that pseudo-genes probably have a higher rate of mutation than 'real' genes. Furthermore, the phenotypic features we address are likely to be plastic, and can be adjusted within individual lifetimes, making them much more changeable than we model here. Thus in reality these phenotypic features are affected by genetic change and phenotypic plasticity. Determining

what is a reasonable mutation rate is therefore difficult, and instead we focus on what different mutation rates reveal about dynamics in our model.
